# Supplementary material for: Speak Up! Simulation Workshop: Teaching Graduate Medical Trainees to Recognize and Respond to Microaggressions in the Clinical Setting
Source: MedEdPORTAL. 2025 Aug 29;21:11545. doi: 10.15766/mep_2374-8265.11545 (PMC12394545; doi:10.15766/mep_2374-8265.11545)
Supplement: Supplementary file 1 — Speak Up! Simulation Workshop - Template.pptxFacilitator Guide and Agenda.docxPostworkshop Survey.docxParticipant Speak Up! Guide.docxDeidentified Microaggression Case Bank.pptx [file mep_2374-8265.11545-s001.zip › C. Postworkshop Survey.docx]

**Instructional use:** Use this survey to evaluate your workshop’s effectiveness by scheduling time into the workshop session at the end and/or emailing participants after the workshop. Surveys can be provided in various formats as preferred (digital form, printed, PDF fill-in, etc.)

Post-workshop Survey

*Thank you for attending Speak Up! Simulation. Please help us assess the impact of our workshop.*

1. Prior to this training, have you ever attended an upstanding training or similar in the clinical setting?
   1. Yes, I've completed one other
   2. Yes, I've completed 2-5
   3. Yes, I've completed 5-10
   4. Yes, I've completed >10
   5. No
2. Have you witnessed an incident of discrimination and/or racism in the clinical setting?
   1. Yes
   2. No
   3. Unsure
3. Prior to this training, have you spoken up against witnessed discrimination and/or racism in the clinical setting? (required)
   1. Yes (go to Q4)
   2. No (go to Q5)
4. What emotions did you associate with the process of speaking up? (check all that apply)
   1. Paralysis/shock
   2. Fearful/Nervousness
   3. Content/Peace
   4. Confusion
   5. Anger
   6. Uncertainty
   7. Pride/confidence
   8. Solidarity
   9. Hurt/Sad
   10. Other: ___________________
5. What factors contributed to your not being able to speak up? (check all that apply)
   1. Delicate therapeutic alliance and/or volatile family encounter
   2. Unsupportive and/or unsafe supervisor
   3. Unsupportive and/or unsafe non-supervisory staff
   4. Internal discomfort and/or avoidance of conflict
   5. Loss of words and/or paralysis
   6. Not worth the effort
   7. Too busy
   8. Forgot
   9. Have never witnessed a microaggression
   10. Other: ______
6. Before the workshop, rate your level of confidence in identifying microaggressions?

| 1 | 2 | 3 | 4 | 5 |
| --- | --- | --- | --- | --- |
| Very unconfident | Unconfident | Neutral | Confident | Very confident |

1. After the workshop, rate your level of confidence in identifying microaggressions?

| 1 | 2 | 3 | 4 | 5 |
| --- | --- | --- | --- | --- |
| Very unconfident | Unconfident | Neutral | Confident | Very confident |

1. Has your comfort of speaking up to address microaggressions changed?
   1. It increased after the workshop (go to Q9)
   2. It decreased after the workshop (go to Q10)
   3. Did not change (go to Q11)
2. What aspect(s) of this workshop contributed to your increase in comfort? (free text)
3. What aspect(s) of this workshop contributed to your decrease in comfort? (free text)
4. Before the workshop, if you witnessed a Black or Brown physician being mistaken for a maintenance worker, what was your likelihood of speaking up?

| 1 | 2 | 3 | 4 | 5 |
| --- | --- | --- | --- | --- |
| Very unlikely to speak up | Unlikely to speak up | Unsure | Likely to speak up | Very likely to speak up |

1. After the workshop, if you were to witness a Black or Brown physician mistaken for a maintenance worker, what is your likelihood of speaking up?

| 1 | 2 | 3 | 4 | 5 |
| --- | --- | --- | --- | --- |
| Very unlikely to speak up | Unlikely to speak up | Unsure | Likely to speak up | Very likely to speak up |

1. Did you feel psychologically safe during the session? Any suggestions for improvement? (free text) Note: consider separating into two questions, with nominal answer choices for the first question and free text for the second.
2. Additional comments: ________________
3. What training program are you in? (free text)
4. What is your current level of graduate medical training?
   1. PGY-1, intern
   2. PGY-2, I senior medical students
   3. PGY-3 or above, I senior residents (PGY-1 or 2)
   4. Fellow - first year
   5. Fellow- senior

*Your responses are valuable to our efforts. We are so grateful for the time and thought you put towards answering this survey. Please don't hesitate to reach out if you need support in the meantime!*
