# Supplementary material for: Speak Up! Simulation Workshop: Teaching Graduate Medical Trainees to Recognize and Respond to Microaggressions in the Clinical Setting
Source: MedEdPORTAL. 2025 Aug 29;21:11545. doi: 10.15766/mep_2374-8265.11545 (PMC12394545; doi:10.15766/mep_2374-8265.11545)
Supplement: Supplementary file 1 — Speak Up! Simulation Workshop - Template.pptxFacilitator Guide and Agenda.docxPostworkshop Survey.docxParticipant Speak Up! Guide.docxDeidentified Microaggression Case Bank.pptx [file mep_2374-8265.11545-s001.zip › D. Participant Speak Up! Guide.docx]

**Instructional use:** Please provide this 1-page guide for each participant to use during each workshop. Recommend printing for in-person and a PDF version for virtual participants.

**Participant Speak Up! Guide**

- Review the case:
  - Identify microaggressions and/or missed opportunities to upstand.
- Self-select or designate roles:
  - Should I speak up?
  - What could you say or do?​ (see below)
  - What reaction/response could happen? ​
  - What are the implications to upstanding, either now or later?​
  - If unable to speak up, how else can the impact be acknowledged?
  - How could this experience have been mitigated?​

| **Pause the conversation**  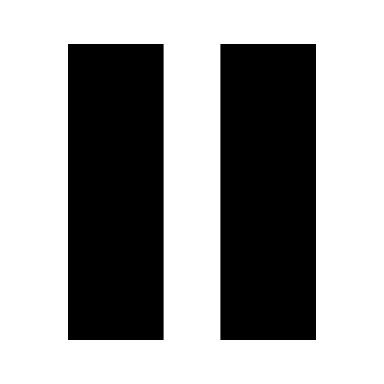 | - Clear your throat, shift your position, or hold up your hand. - “[Before you/we go on], I’d like to acknowledge what happened.” - "Sorry, could you say that again?” |
| --- | --- |
| **Seek clarification**  **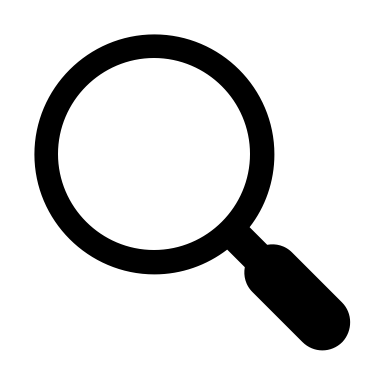** | - “I am curious, what did you mean when you said/did [____]?” - “I’d like to understand, was there something that prompted your comment/question? - “Tell me more.” - “What I heard you say was ____, is that correct?” |
| **Name behavior as inappropriate**  **Set boundaries**  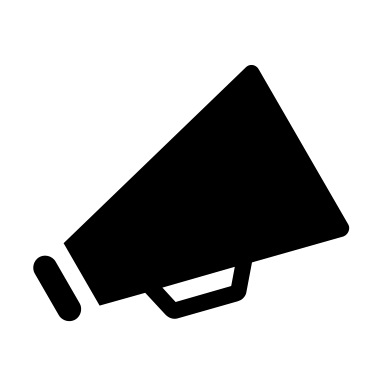 | - “What happened made me feel [____]. - “We don’t use that language here.” - “Let’s keep it professional.” - “That’s not okay.” |
| **Refocus to the professional context**  **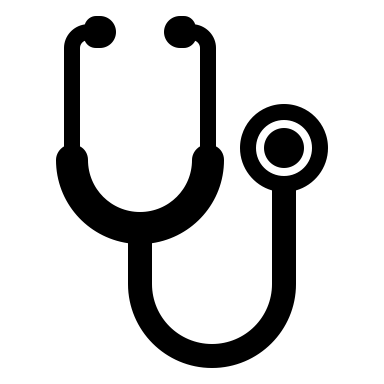** | - “You may have been trying to compliment/joke, but let’s get back to why we are here/what we are here for.” - “I am willing to come back and discuss this further with you, but for now let’s focus on________.” - “Acknowledging what happened, even though it’s uncomfortable for everyone, will help us all move forward in your care/with our jobs.” |
| ***Value the person***  ***Validate worth***  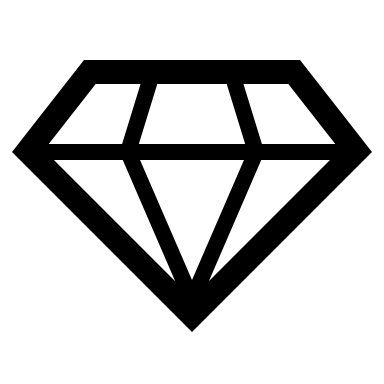 | - “I respect every patient. I expect you do as well.” - “___ is one of my learners/supervisors. I do not appreciate anyone treating my learner/supervisor this way.” - “I agree with ____, what happened was not appropriate or deserved, no matter how benign it seemed.” |

Adapted from: Oluo, I. (2019). *So you want to talk about race*. Seal Press.
