# Supplementary material for: Speak Up! Simulation Workshop: Teaching Graduate Medical Trainees to Recognize and Respond to Microaggressions in the Clinical Setting
Source: MedEdPORTAL. 2025 Aug 29;21:11545. doi: 10.15766/mep_2374-8265.11545 (PMC12394545; doi:10.15766/mep_2374-8265.11545)
Supplement: Supplementary file 1 — Speak Up! Simulation Workshop - Template.pptxFacilitator Guide and Agenda.docxPostworkshop Survey.docxParticipant Speak Up! Guide.docxDeidentified Microaggression Case Bank.pptx [file mep_2374-8265.11545-s001.zip › E. Deidentified Microaggression Case Bank.pptx]

## Slide 1
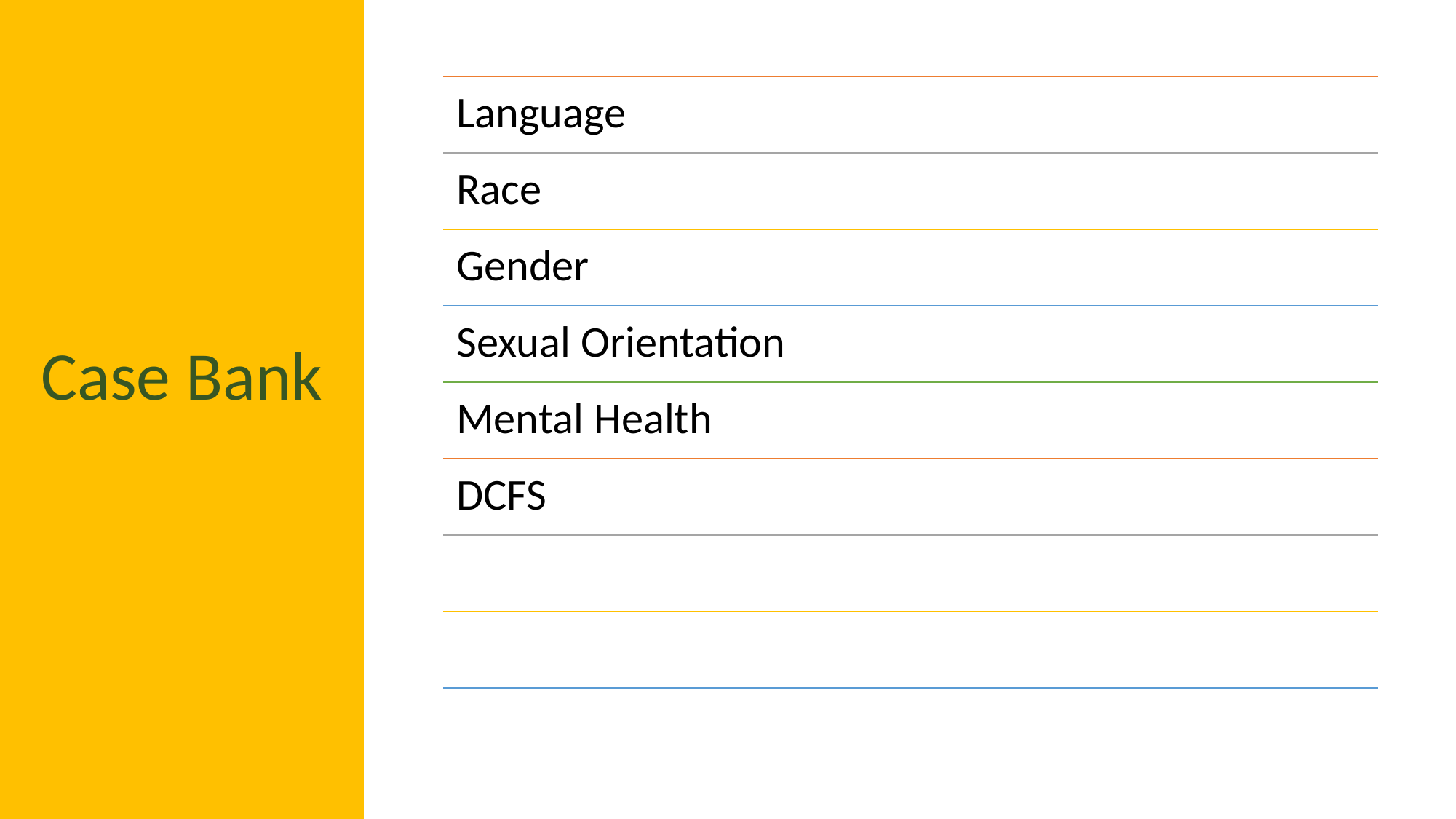

# Case Bank
Language
Race
Gender
Sexual Orientation
Mental Health
DCFS

## Slide 2
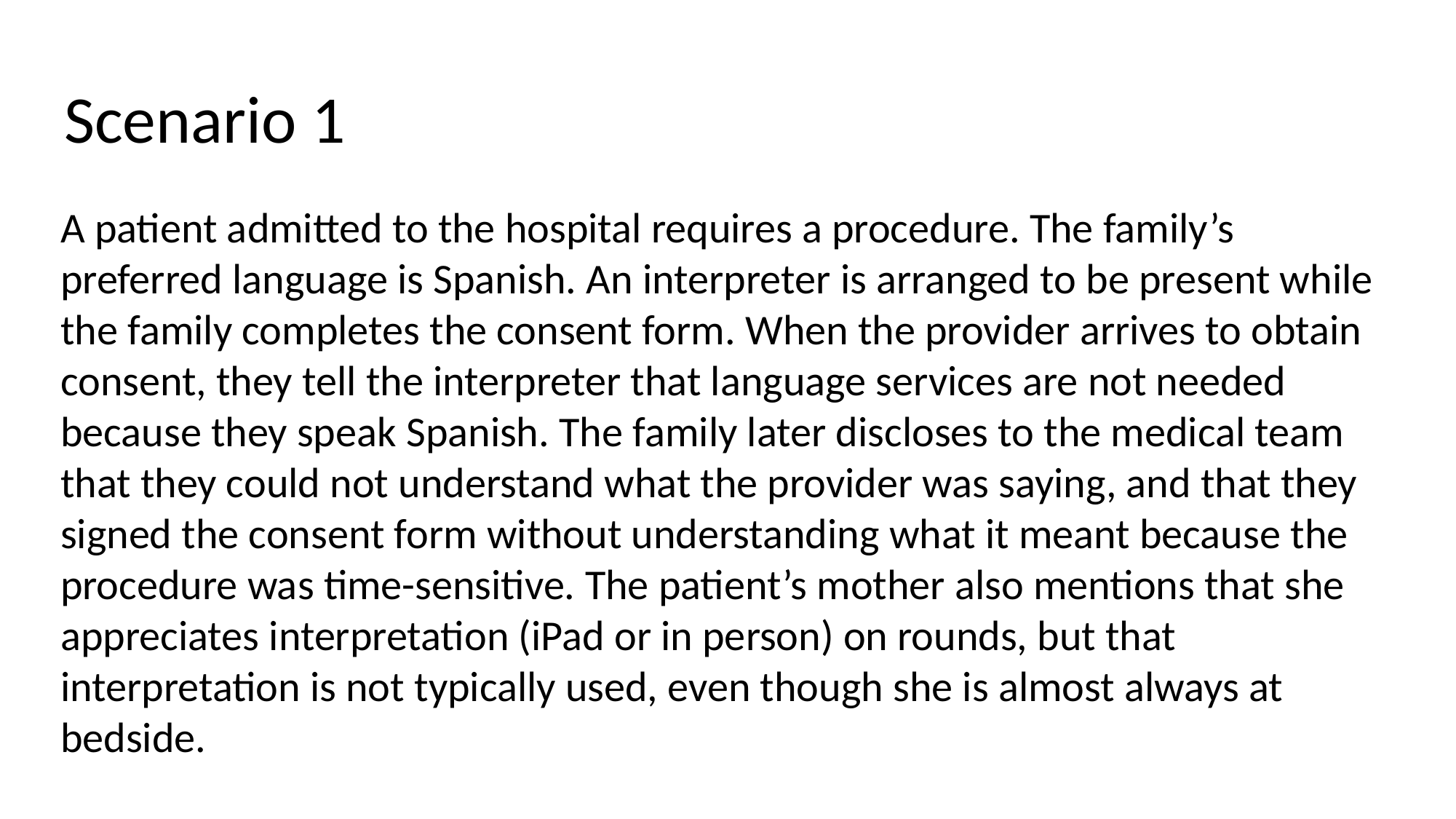

# Scenario 1
A patient admitted to the hospital requires a procedure. The family’s preferred language is Spanish. An interpreter is arranged to be present while the family completes the consent form. When the provider arrives to obtain consent, they tell the interpreter that language services are not needed because they speak Spanish. The family later discloses to the medical team that they could not understand what the provider was saying, and that they signed the consent form without understanding what it meant because the procedure was time-sensitive. The patient’s mother also mentions that she appreciates interpretation (iPad or in person) on rounds, but that interpretation is not typically used, even though she is almost always at bedside.

## Slide 3
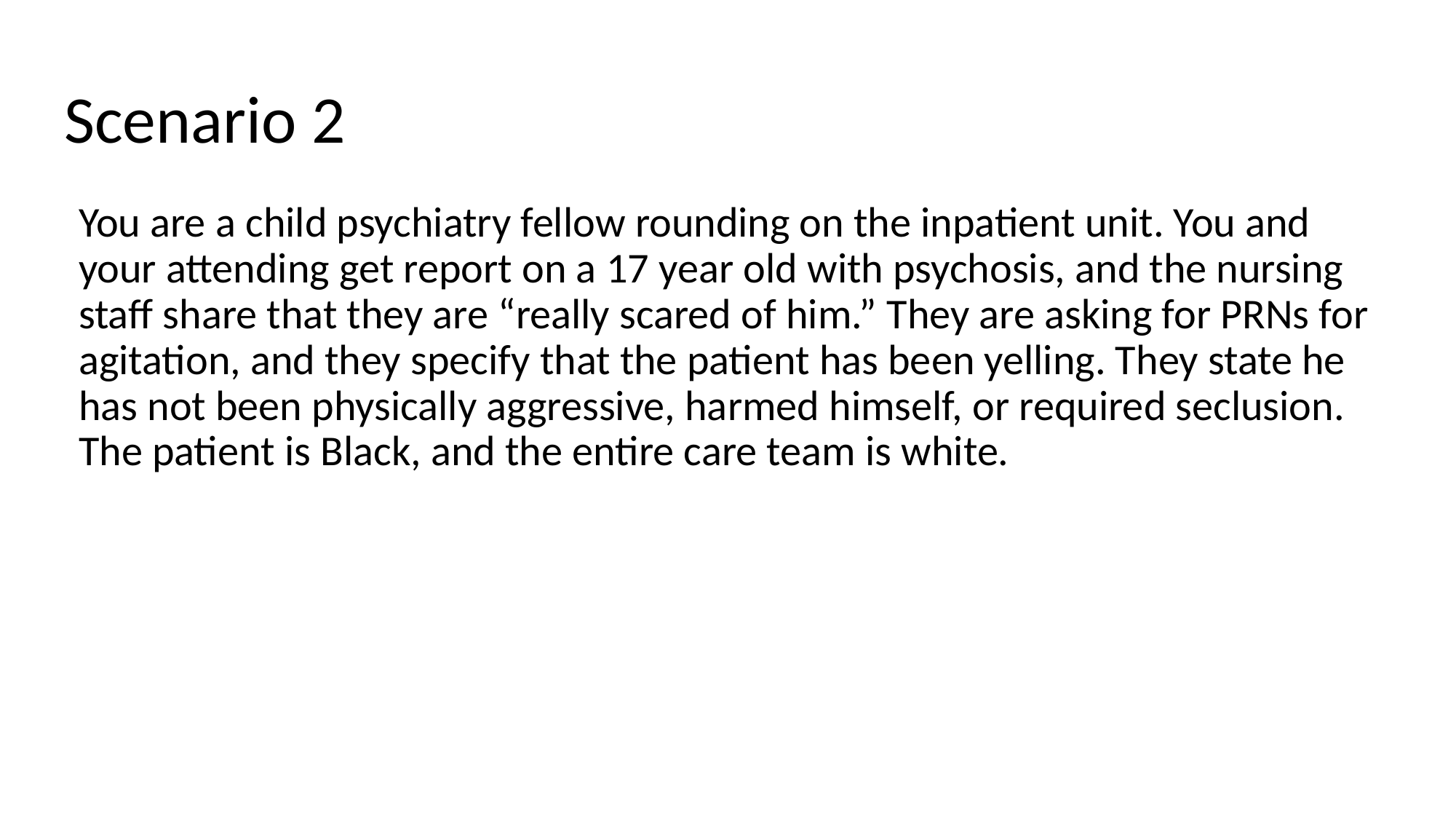

# Scenario 2
You are a child psychiatry fellow rounding on the inpatient unit. You and your attending get report on a 17 year old with psychosis, and the nursing staff share that they are “really scared of him.” They are asking for PRNs for agitation, and they specify that the patient has been yelling. They state he has not been physically aggressive, harmed himself, or required seclusion. The patient is Black, and the entire care team is white.

## Slide 4
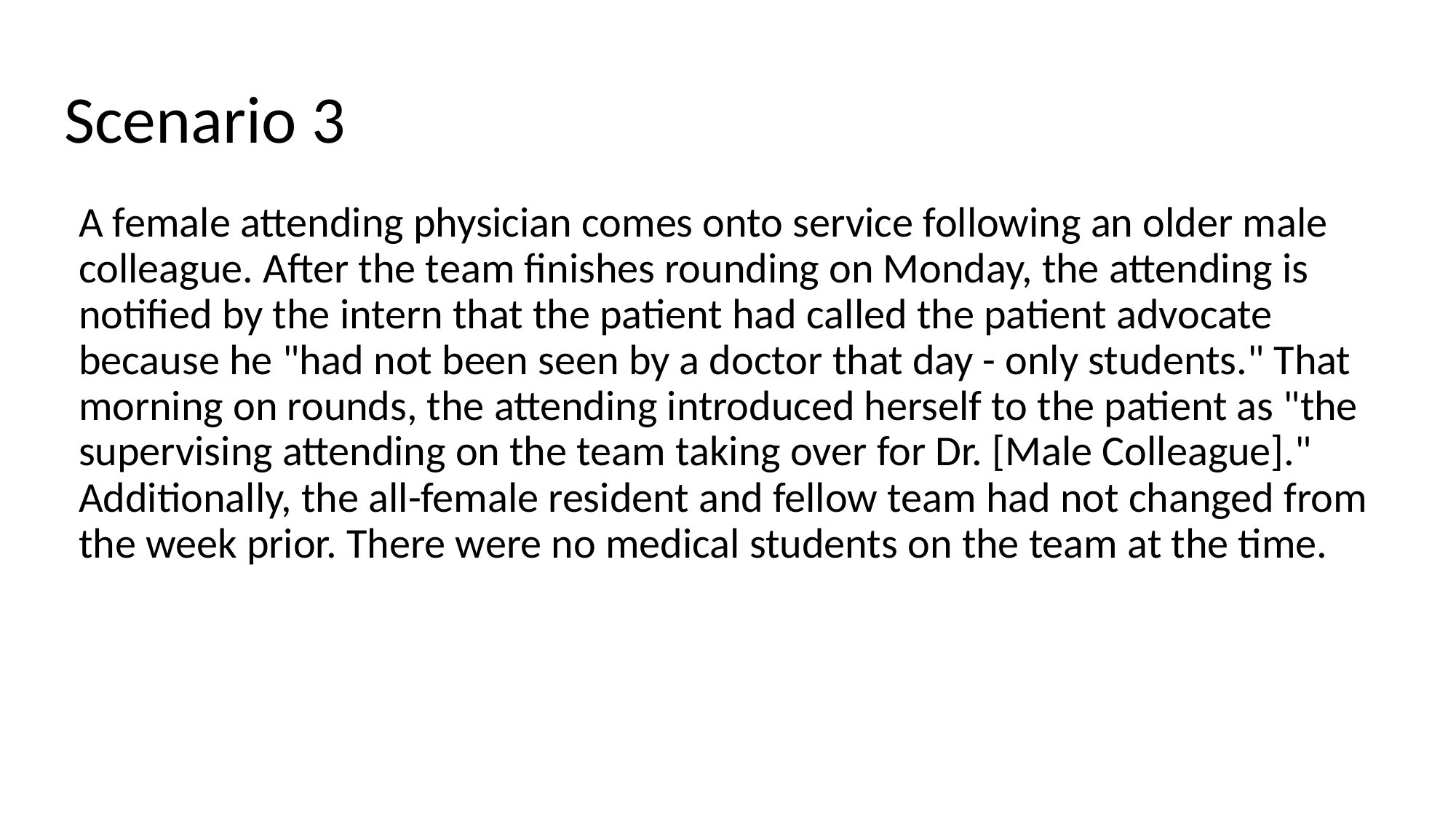

# Scenario 3
A female attending physician comes onto service following an older male colleague. After the team finishes rounding on Monday, the attending is notified by the intern that the patient had called the patient advocate because he "had not been seen by a doctor that day - only students." That morning on rounds, the attending introduced herself to the patient as "the supervising attending on the team taking over for Dr. [Male Colleague]." Additionally, the all-female resident and fellow team had not changed from the week prior. There were no medical students on the team at the time.

## Slide 5
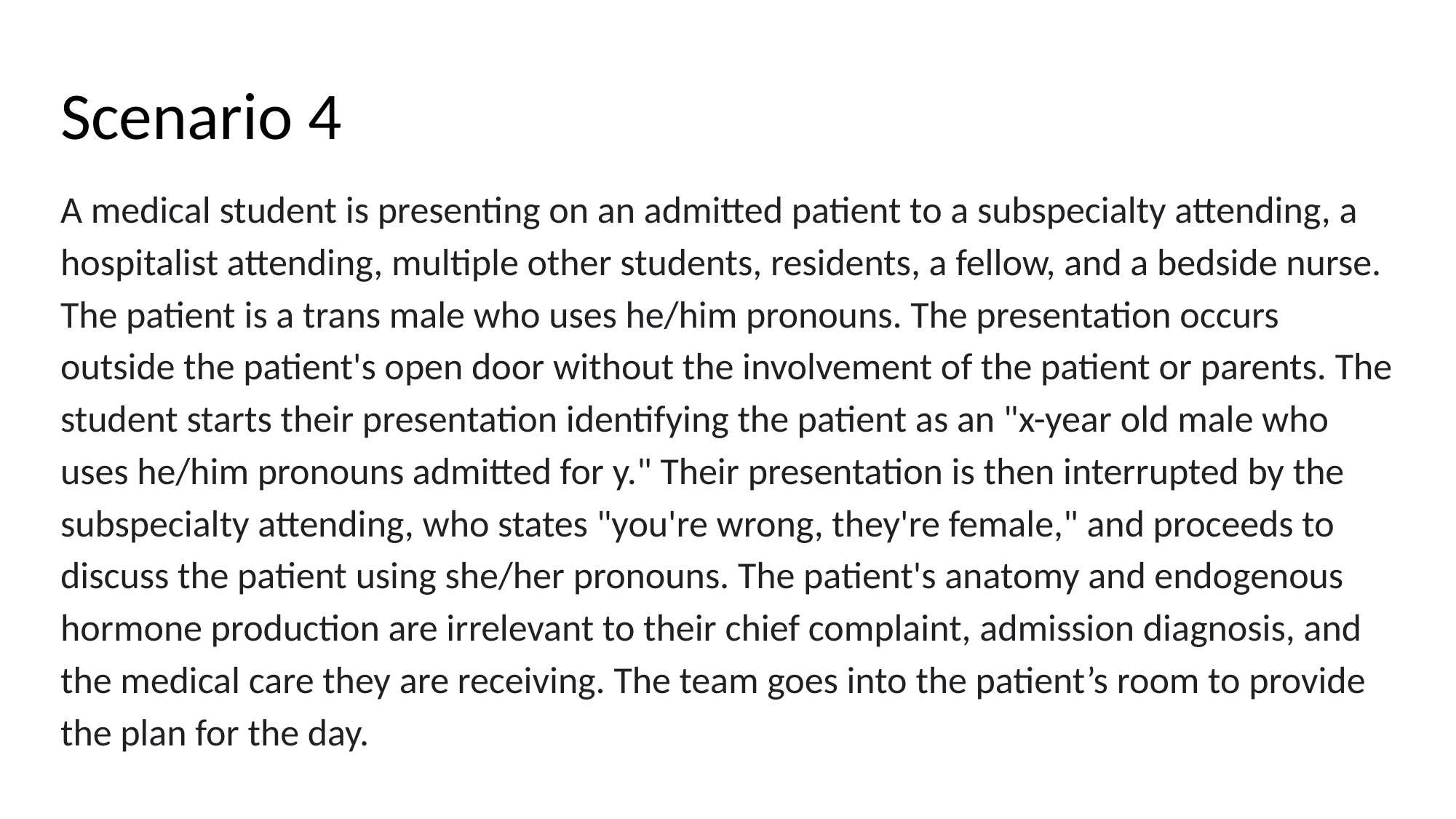

# Scenario 4
A medical student is presenting on an admitted patient to a subspecialty attending, a hospitalist attending, multiple other students, residents, a fellow, and a bedside nurse. The patient is a trans male who uses he/him pronouns. The presentation occurs outside the patient's open door without the involvement of the patient or parents. The student starts their presentation identifying the patient as an "x-year old male who uses he/him pronouns admitted for y." Their presentation is then interrupted by the subspecialty attending, who states "you're wrong, they're female," and proceeds to discuss the patient using she/her pronouns. The patient's anatomy and endogenous hormone production are irrelevant to their chief complaint, admission diagnosis, and the medical care they are receiving. The team goes into the patient’s room to provide the plan for the day.

## Slide 6
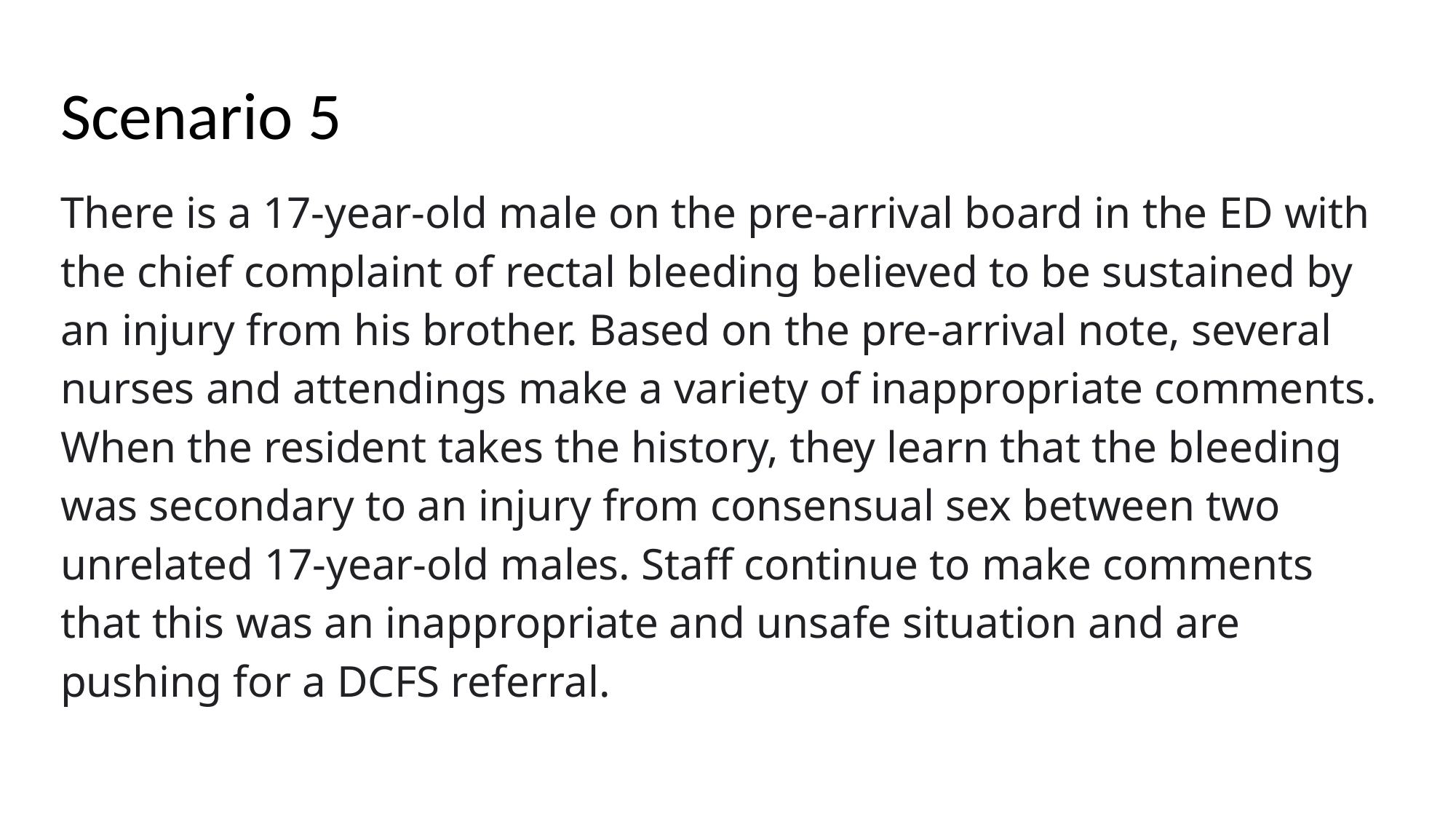

# Scenario 5
There is a 17-year-old male on the pre-arrival board in the ED with the chief complaint of rectal bleeding believed to be sustained by an injury from his brother. Based on the pre-arrival note, several nurses and attendings make a variety of inappropriate comments. When the resident takes the history, they learn that the bleeding was secondary to an injury from consensual sex between two unrelated 17-year-old males. Staff continue to make comments that this was an inappropriate and unsafe situation and are pushing for a DCFS referral.

## Slide 7
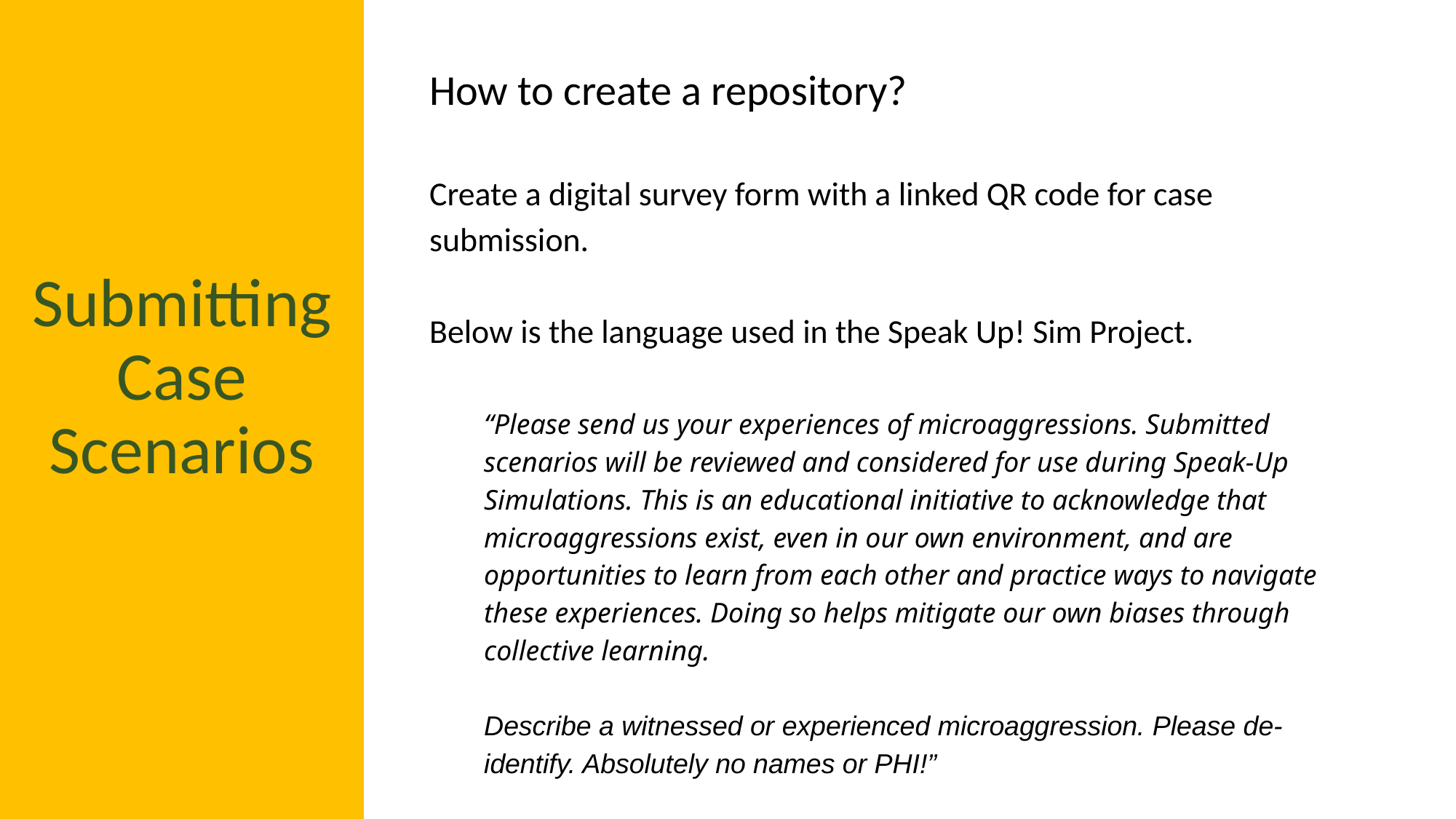

How to create a repository?
Create a digital survey form with a linked QR code for case submission.
Below is the language used in the Speak Up! Sim Project.
“Please send us your experiences of microaggressions. Submitted scenarios will be reviewed and considered for use during Speak-Up Simulations. This is an educational initiative to acknowledge that microaggressions exist, even in our own environment, and are opportunities to learn from each other and practice ways to navigate these experiences. Doing so helps mitigate our own biases through collective learning.
Describe a witnessed or experienced microaggression. Please de-identify. Absolutely no names or PHI!”
# Submitting Case Scenarios
