# Supplementary material for: Speak Up! Simulation Workshop: Teaching Graduate Medical Trainees to Recognize and Respond to Microaggressions in the Clinical Setting
Source: MedEdPORTAL. 2025 Aug 29;21:11545. doi: 10.15766/mep_2374-8265.11545 (PMC12394545; doi:10.15766/mep_2374-8265.11545)
Supplement: Supplementary file 1 — Speak Up! Simulation Workshop - Template.pptxFacilitator Guide and Agenda.docxPostworkshop Survey.docxParticipant Speak Up! Guide.docxDeidentified Microaggression Case Bank.pptx [file mep_2374-8265.11545-s001.zip › A. Speak Up! Simulation Workshop - Template.pptx]

## Slide 1
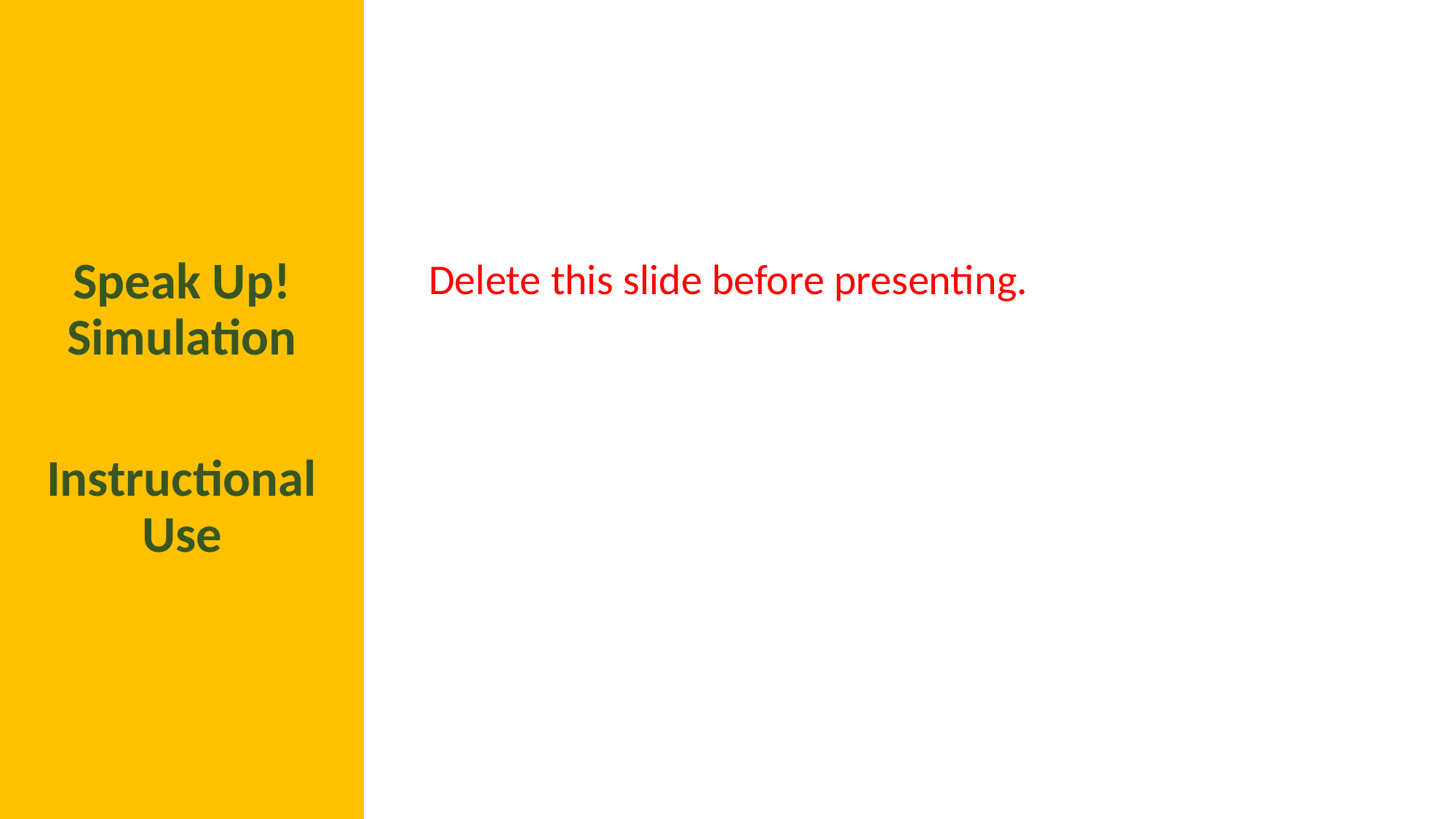

Speak Up! Simulation
Instructional Use
Delete this slide before presenting.

## Slide 2
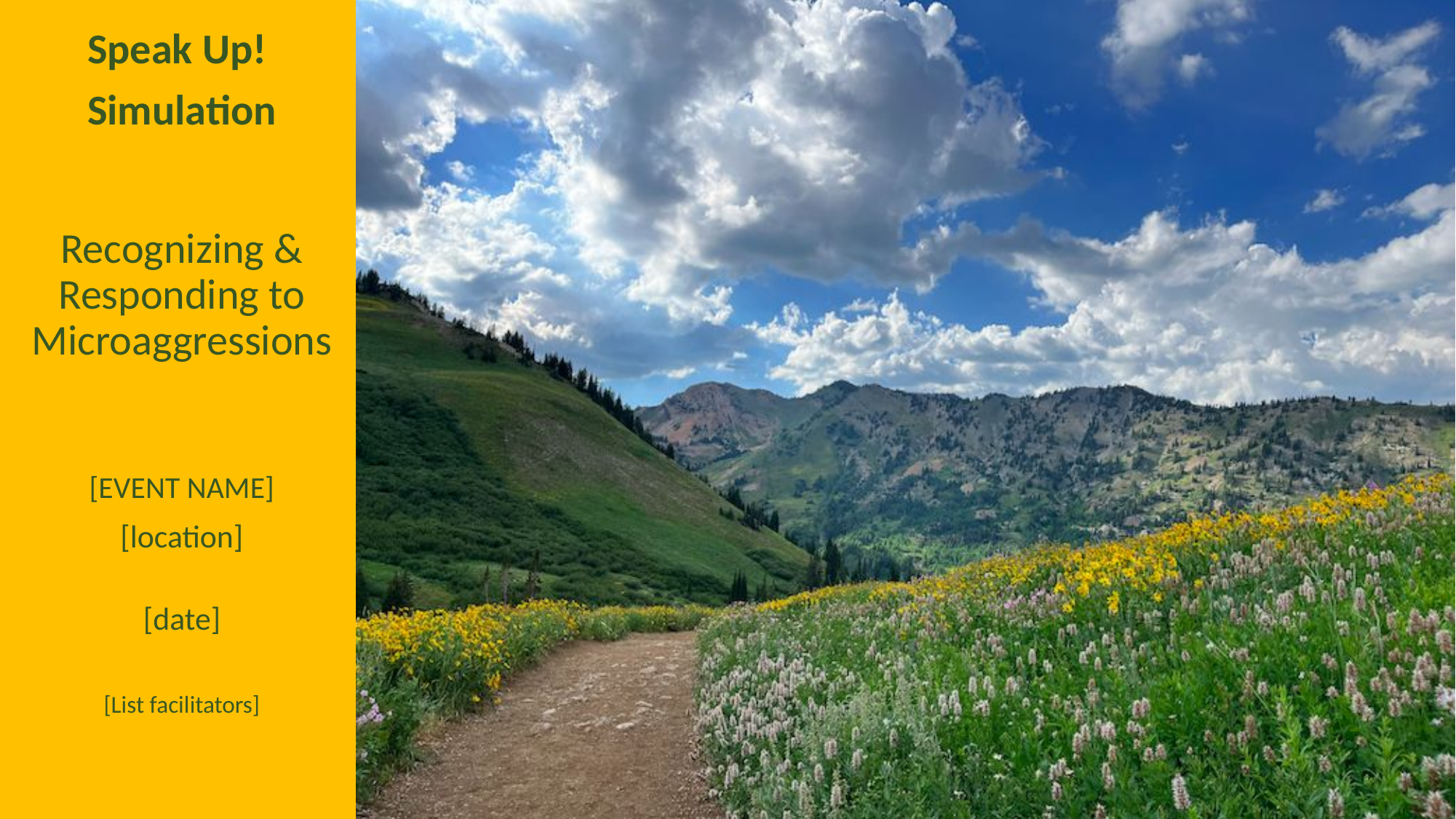

Speak Up!
Simulation
Recognizing & Responding to Microaggressions
[EVENT NAME]
[location]
[date]
[List facilitators]
# Speak Up! Sim

## Slide 3
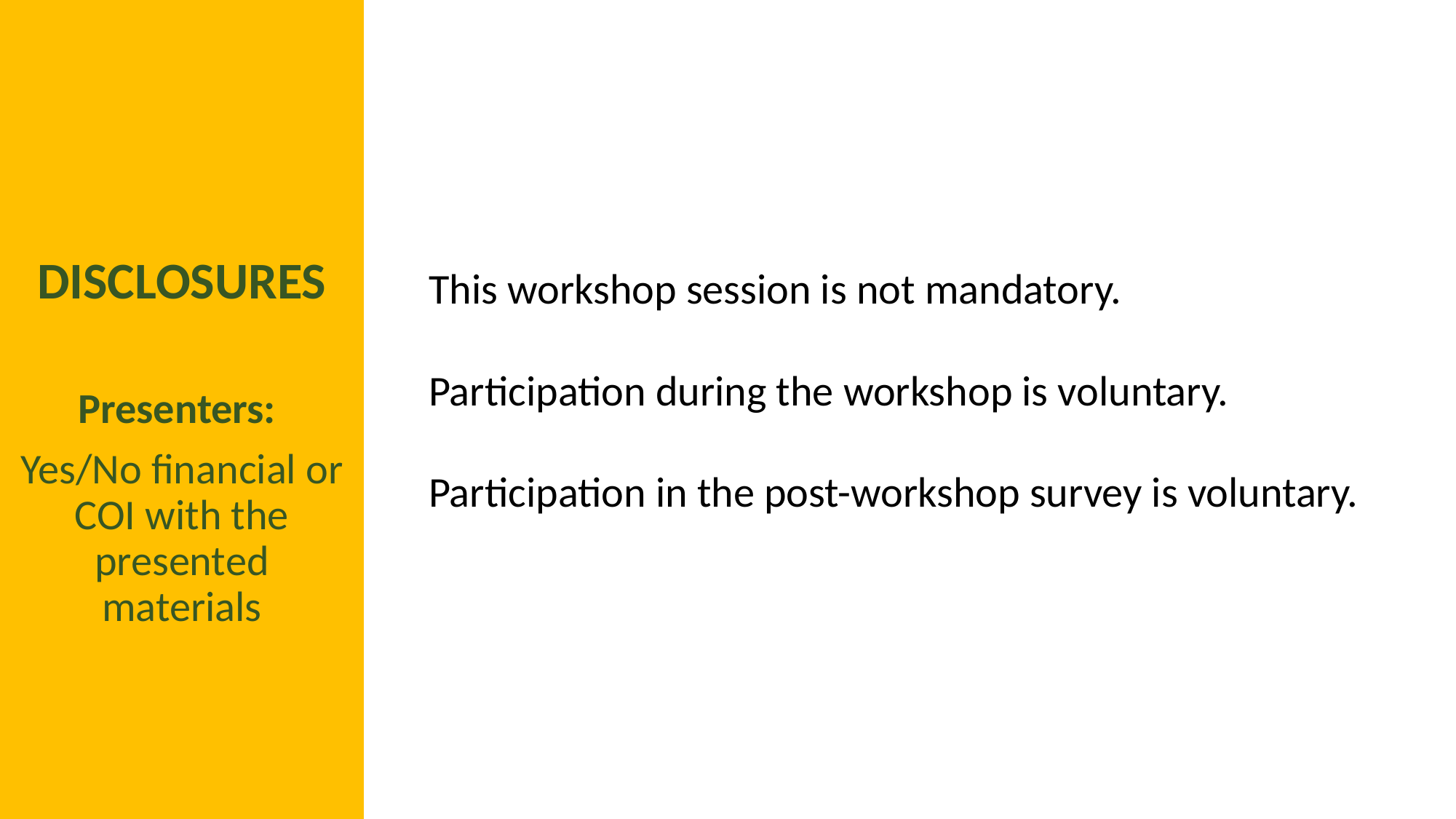

DISCLOSURES
Presenters:
Yes/No financial or COI with the presented materials
This workshop session is not mandatory.
Participation during the workshop is voluntary.
Participation in the post-workshop survey is voluntary.

## Slide 4
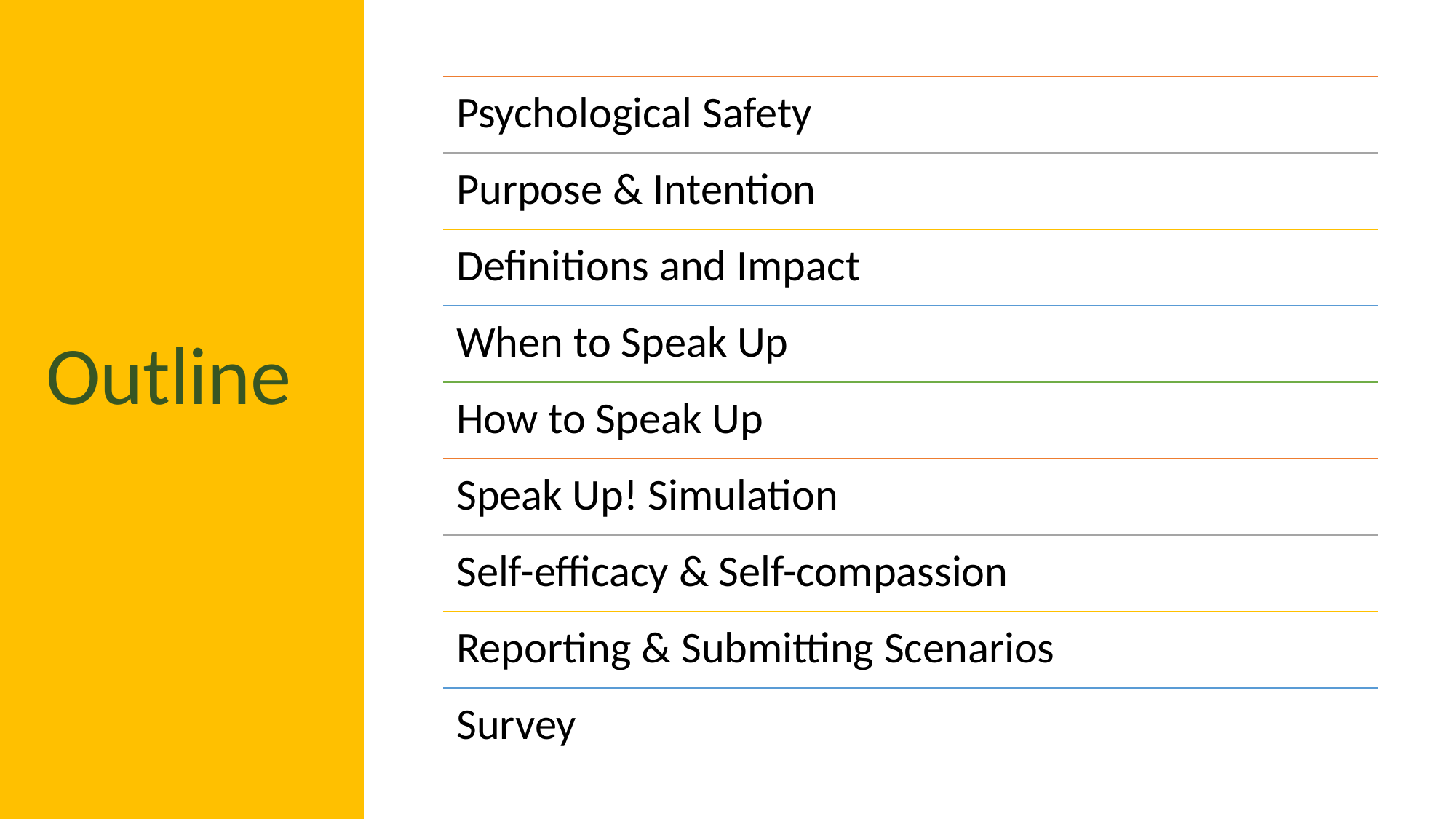

# Outline
Psychological Safety
Purpose & Intention
Definitions and Impact
When to Speak Up
How to Speak Up
Speak Up! Simulation
Self-efficacy & Self-compassion
Reporting & Submitting Scenarios
Survey

## Slide 5
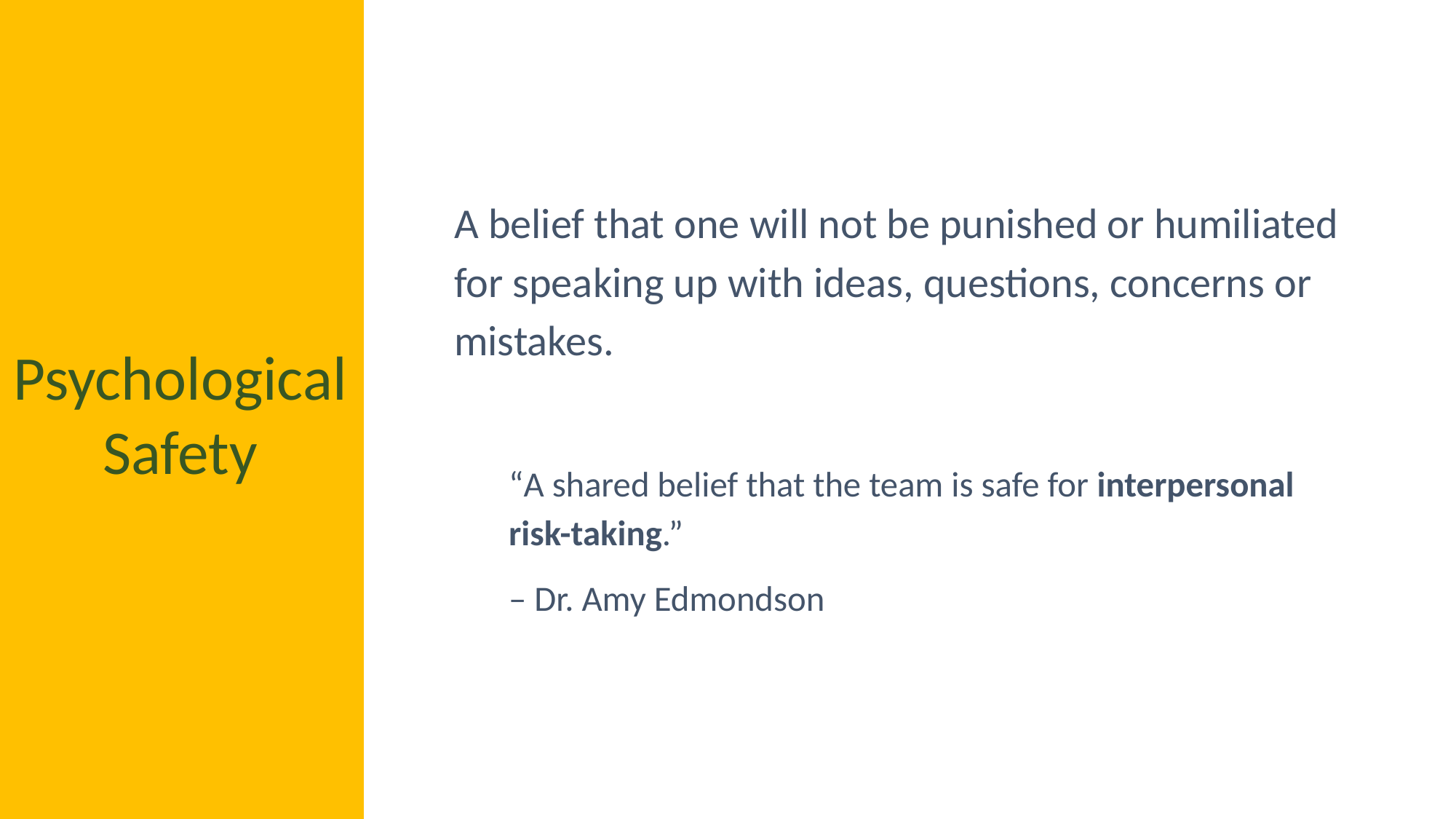

A belief that one will not be punished or humiliated for speaking up with ideas, questions, concerns or mistakes.
“A shared belief that the team is safe for interpersonal risk-taking.”
– Dr. Amy Edmondson
Psychological Safety

## Slide 6
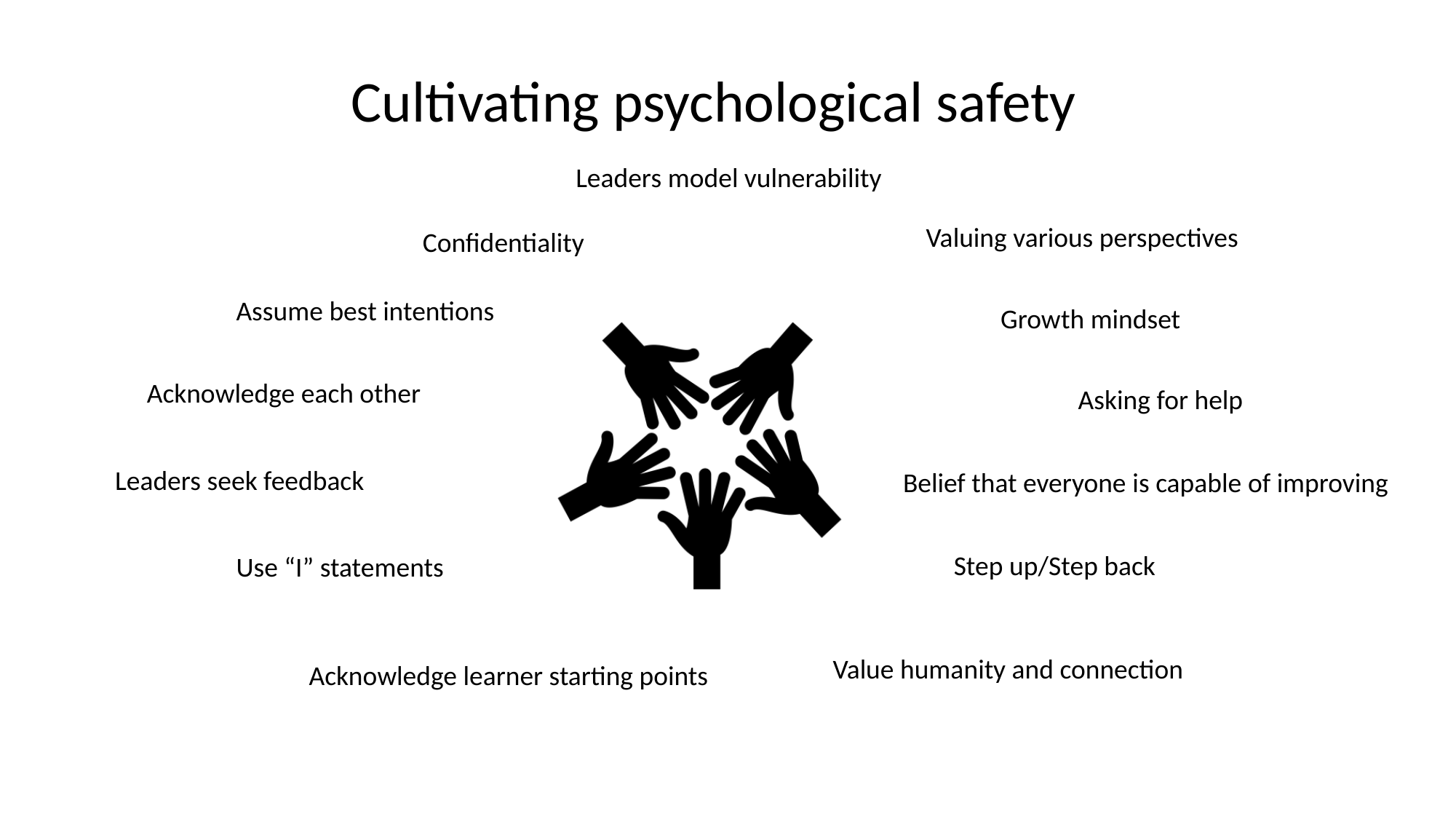

# Cultivating psychological safety
Leaders model vulnerability
Valuing various perspectives
Confidentiality
Assume best intentions
Growth mindset
Acknowledge each other
Asking for help
Leaders seek feedback
Belief that everyone is capable of improving ​​
Step up/Step back
Use “I” statements
Value humanity and connection
Acknowledge learner starting points​

## Slide 7
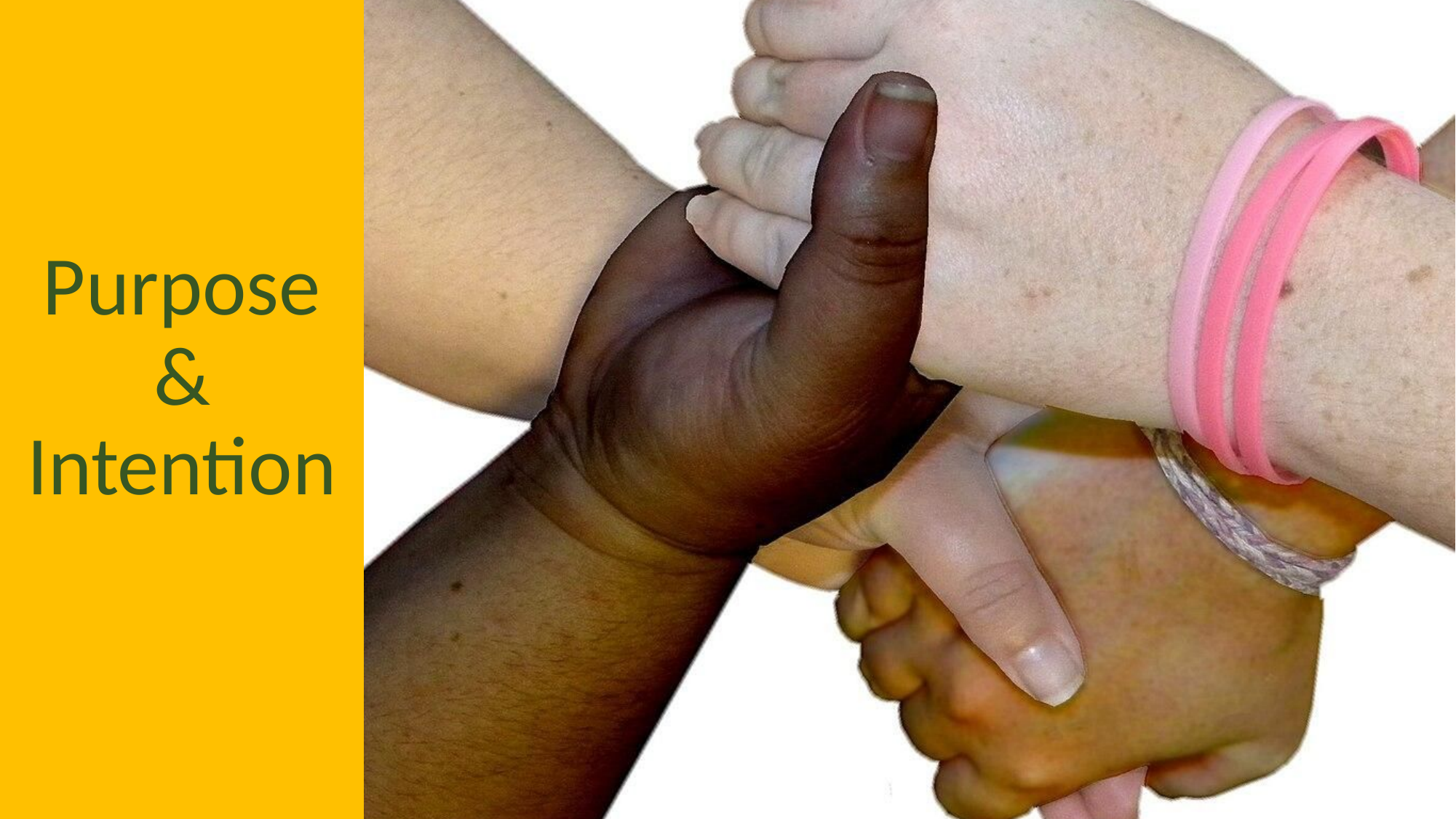

Purpose & Intention

## Slide 8
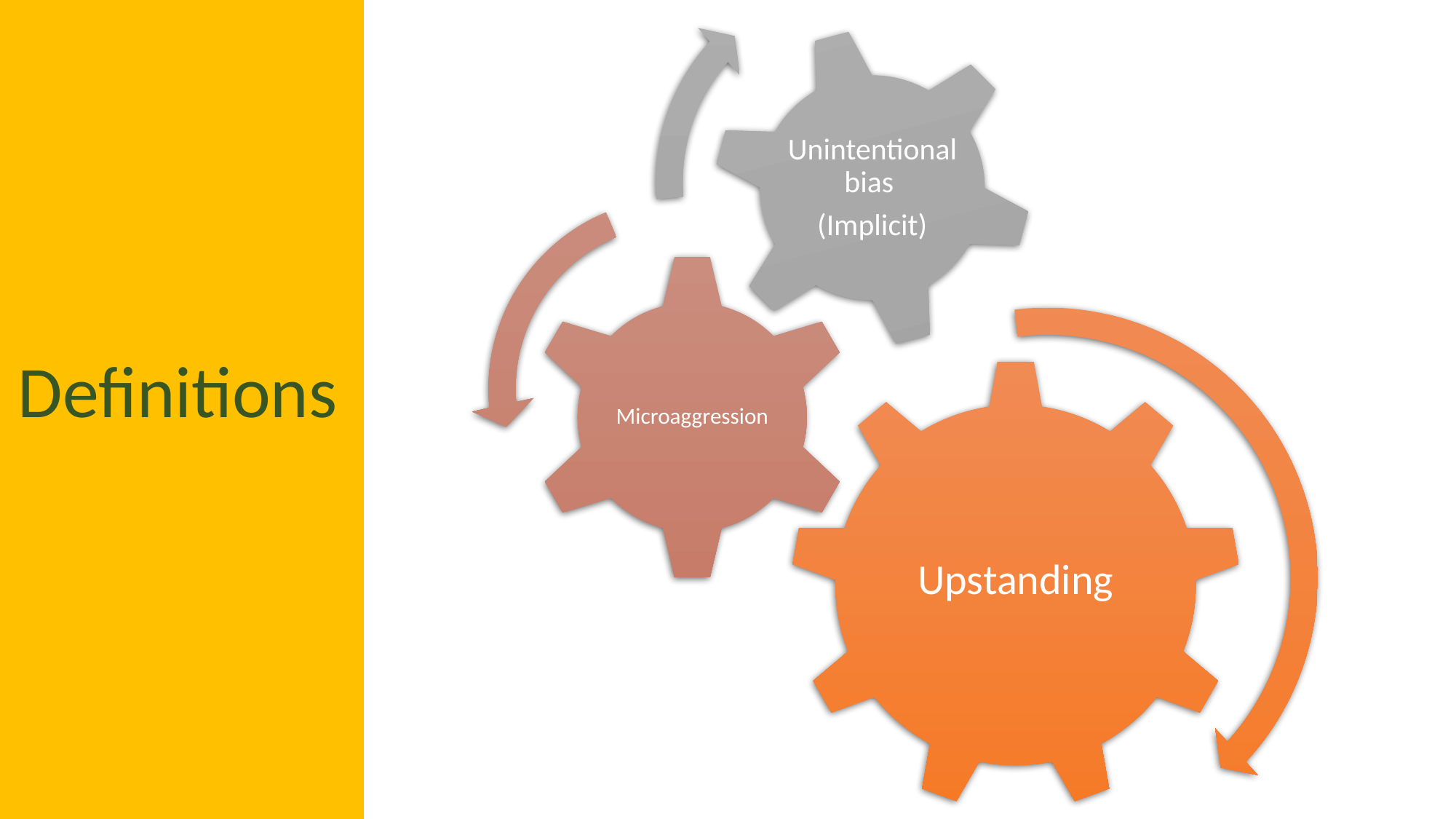

Unintentional bias
(Implicit)
Microaggression
Upstanding
Definitions

## Slide 9
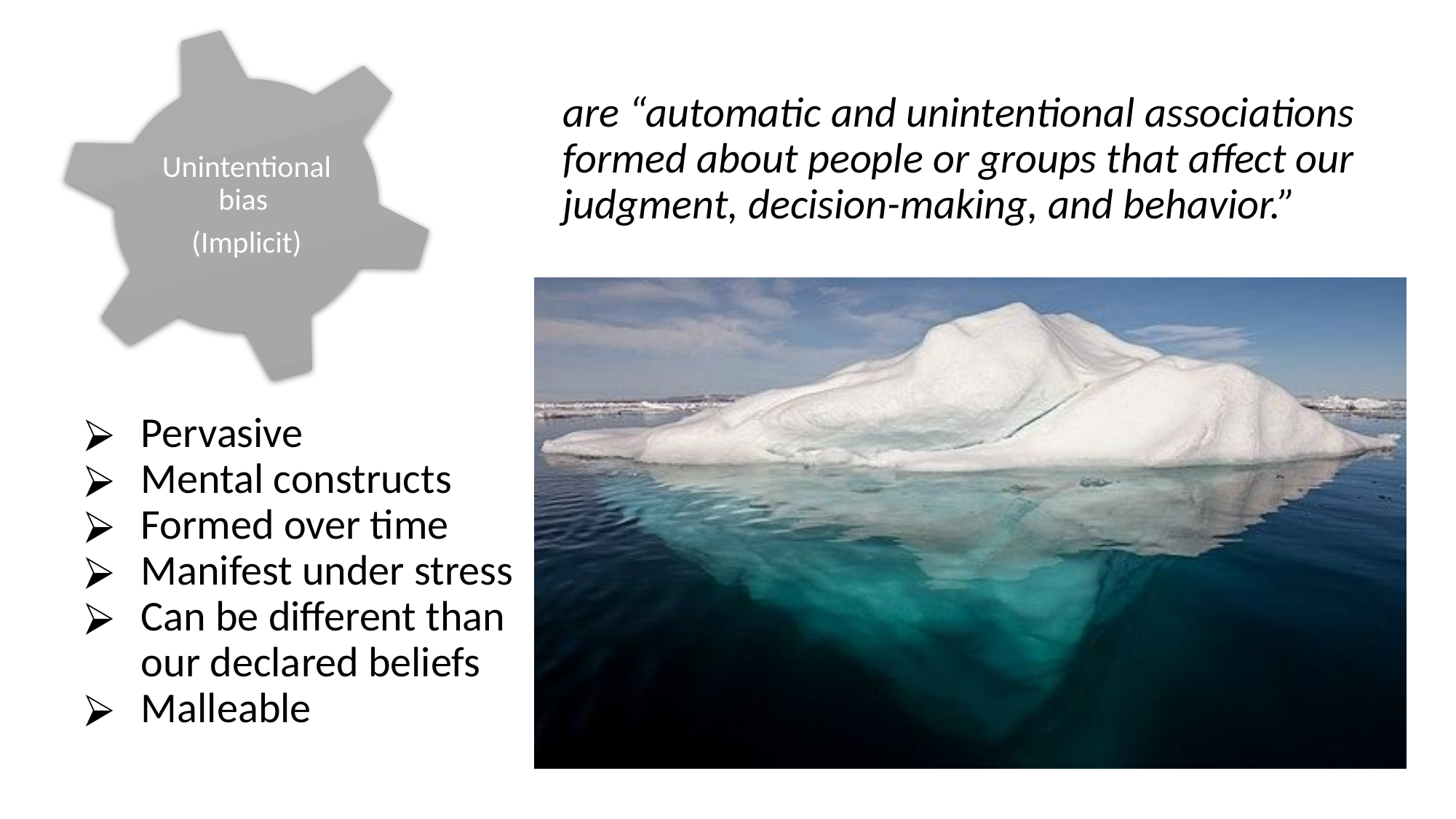

are “automatic and unintentional associations formed about people or groups that affect our judgment, decision-making, and behavior.”
Unintentional bias
(Implicit)
Pervasive
Mental constructs
Formed over time
Manifest under stress
Can be different than our declared beliefs
Malleable

## Slide 10
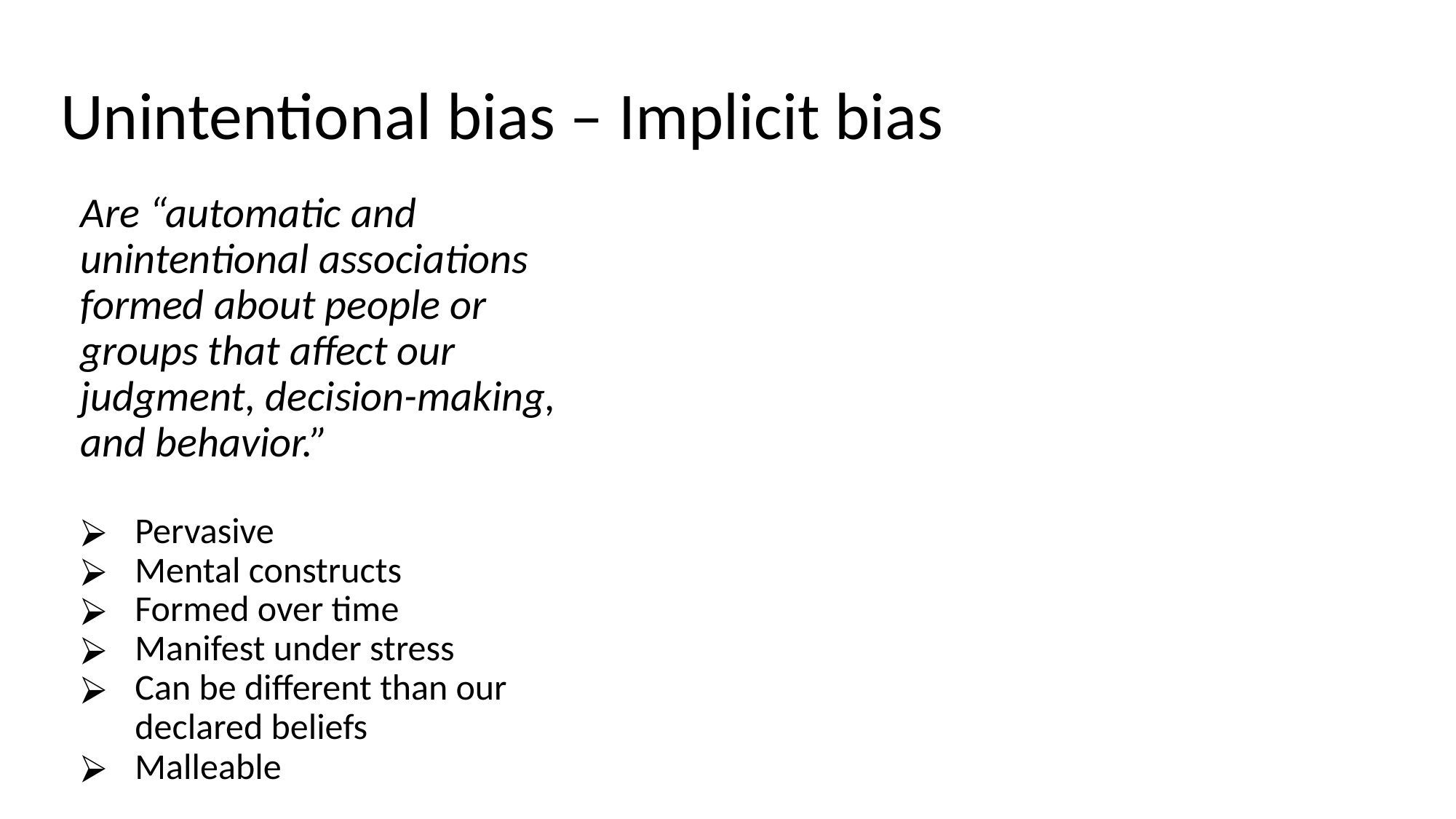

# Unintentional bias – Implicit bias
Are “automatic and unintentional associations formed about people or groups that affect our judgment, decision-making, and behavior.”
Pervasive
Mental constructs
Formed over time
Manifest under stress
Can be different than our declared beliefs
Malleable

## Slide 11
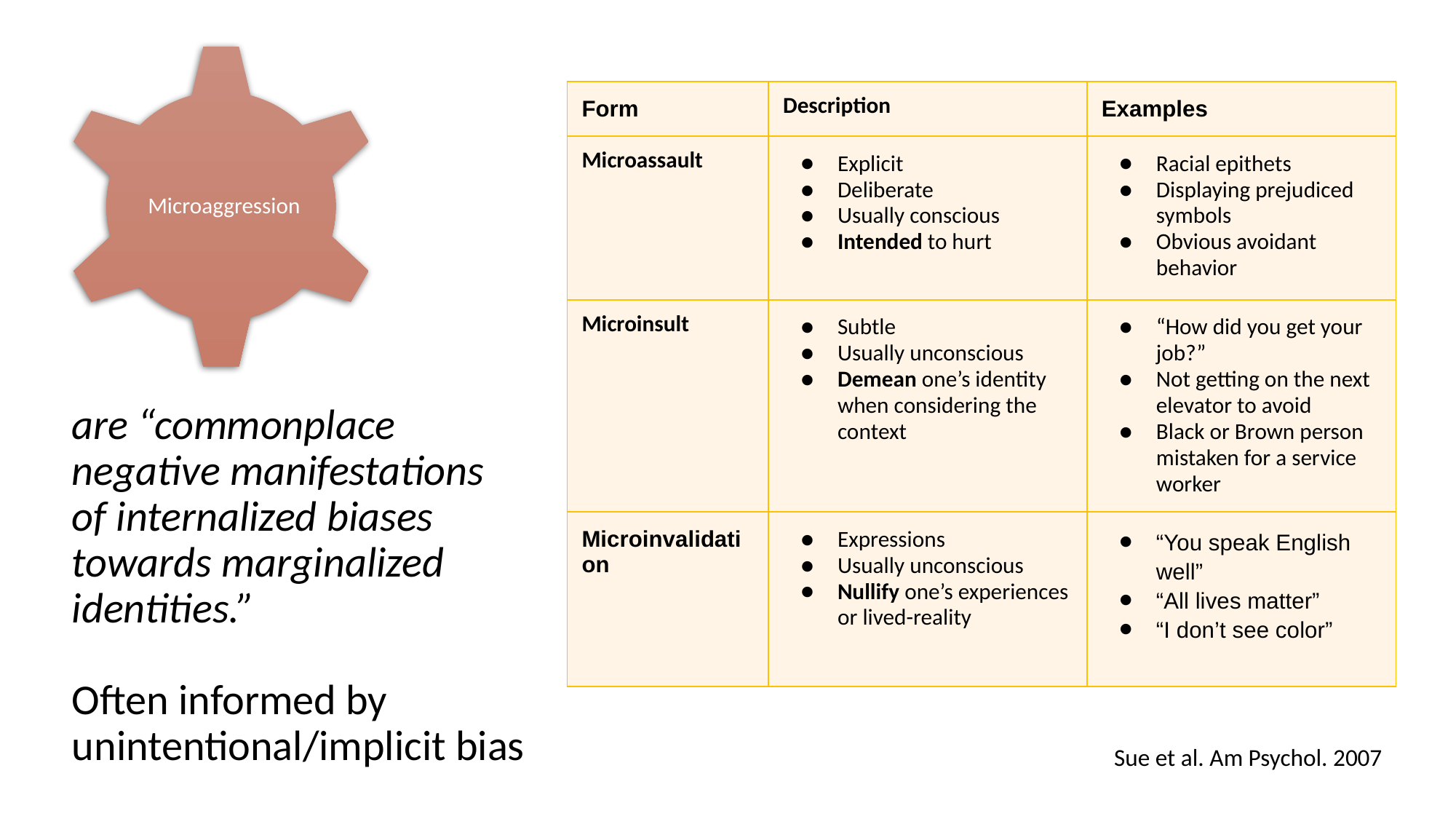

| Form | Description | Examples |
| --- | --- | --- |
| Microassault | Explicit Deliberate Usually conscious Intended to hurt | Racial epithets Displaying prejudiced symbols Obvious avoidant behavior |
| Microinsult | Subtle Usually unconscious Demean one’s identity when considering the context | “How did you get your job?” Not getting on the next elevator to avoid Black or Brown person mistaken for a service worker |
| Microinvalidation | Expressions Usually unconscious Nullify one’s experiences or lived-reality | “You speak English well” “All lives matter” “I don’t see color” |
Microaggression
are “commonplace negative manifestations of internalized biases towards marginalized identities.”
Often informed by unintentional/implicit bias
Sue et al. Am Psychol. 2007

## Slide 12
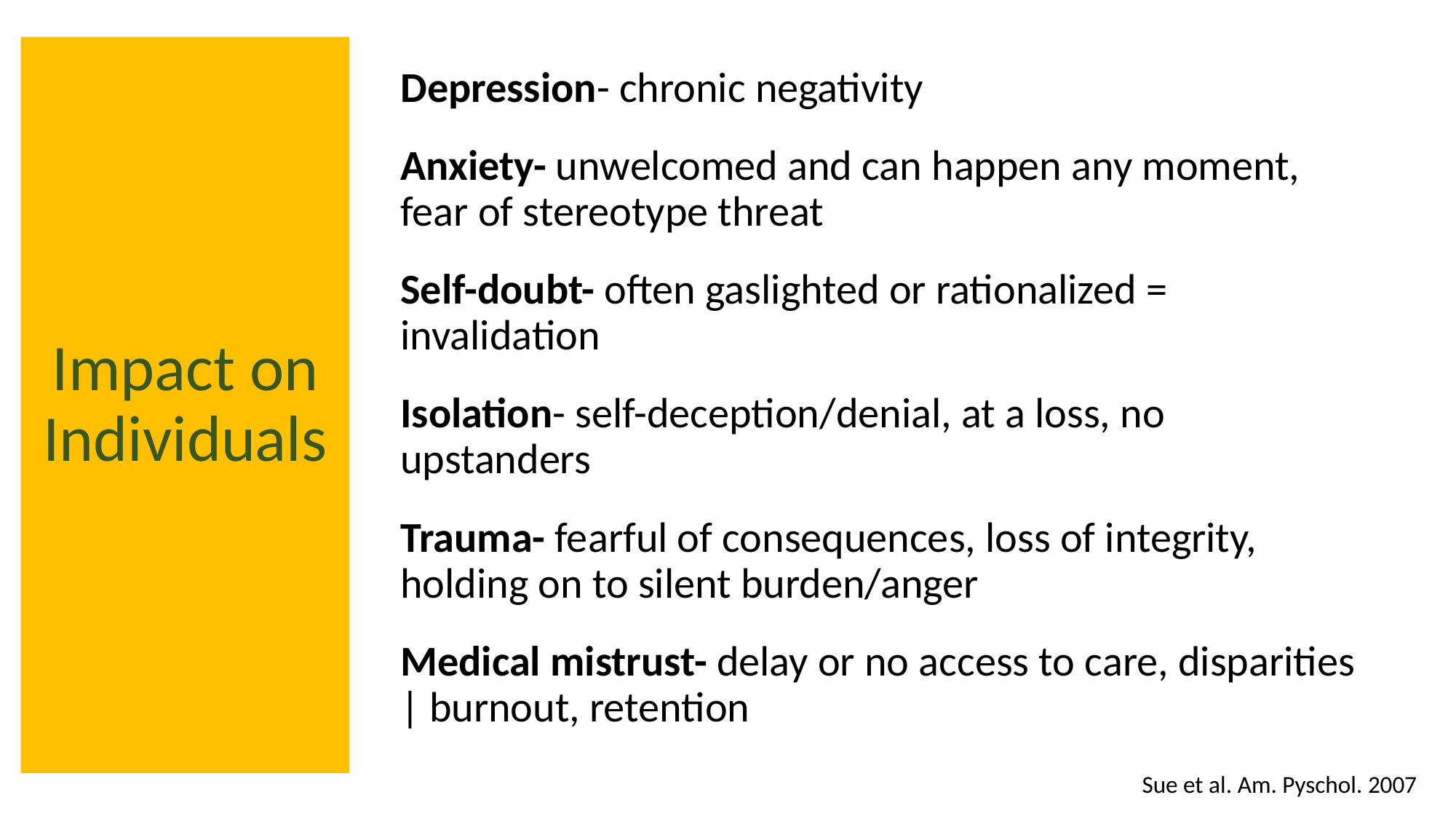

Impact on Individuals
Depression- chronic negativity
Anxiety- unwelcomed and can happen any moment, fear of stereotype threat
Self-doubt- often gaslighted or rationalized = invalidation
Isolation- self-deception/denial, at a loss, no upstanders
Trauma- fearful of consequences, loss of integrity, holding on to silent burden/anger
Medical mistrust- delay or no access to care, disparities | burnout, retention
Sue et al. Am. Pyschol. 2007

## Slide 13
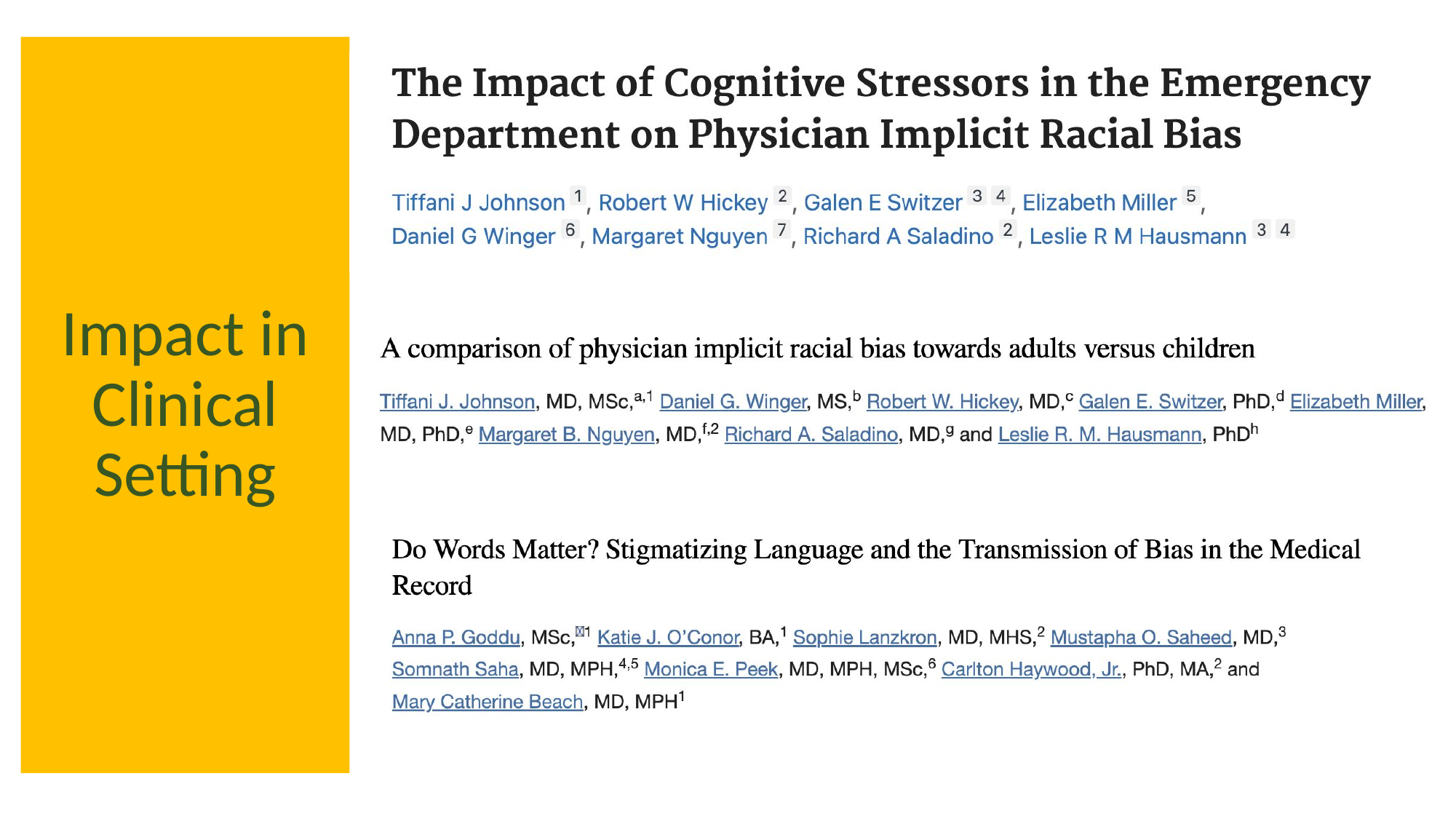

Impact in Clinical Setting

## Slide 14
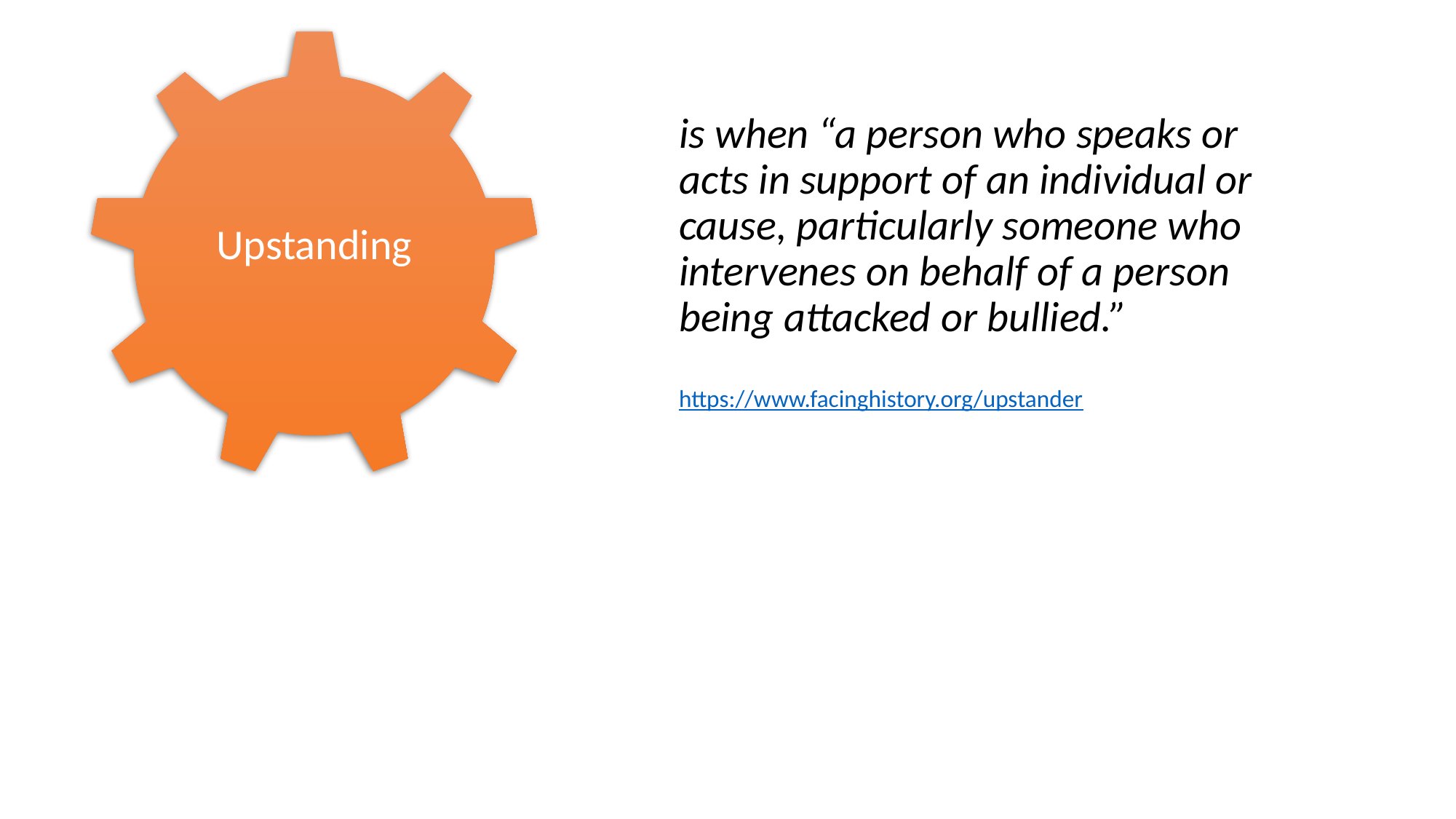

is when “a person who speaks or acts in support of an individual or cause, particularly someone who intervenes on behalf of a person being attacked or bullied.”
https://www.facinghistory.org/upstander
Upstanding

## Slide 15
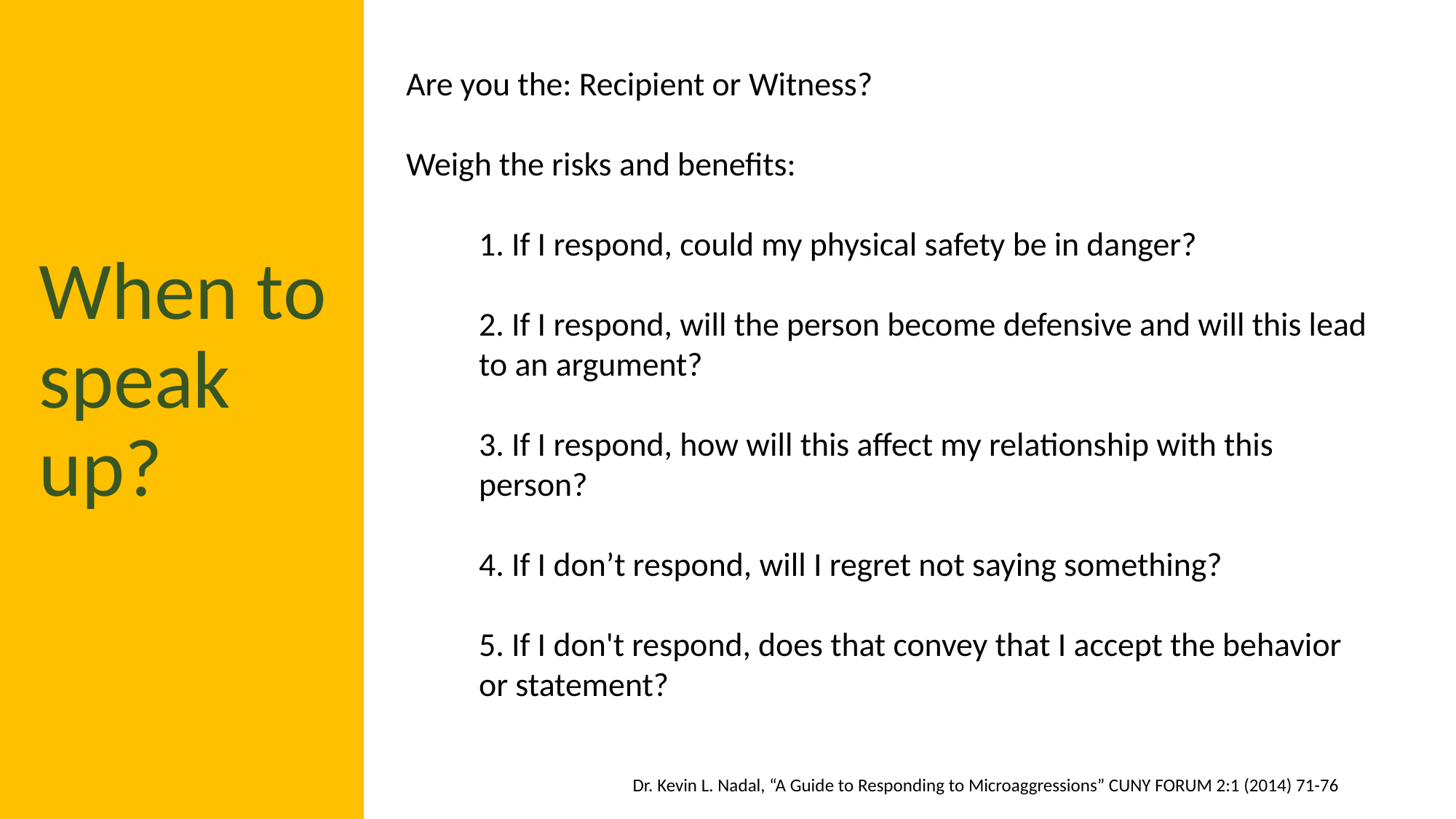

Are you the: Recipient or Witness?
Weigh the risks and benefits:
1. If I respond, could my physical safety be in danger?
2. If I respond, will the person become defensive and will this lead to an argument?
3. If I respond, how will this affect my relationship with this person?
4. If I don’t respond, will I regret not saying something?
5. If I don't respond, does that convey that I accept the behavior or statement?
When to speak up?
Dr. Kevin L. Nadal, “A Guide to Responding to Microaggressions” CUNY FORUM 2:1 (2014) 71-76

## Slide 16
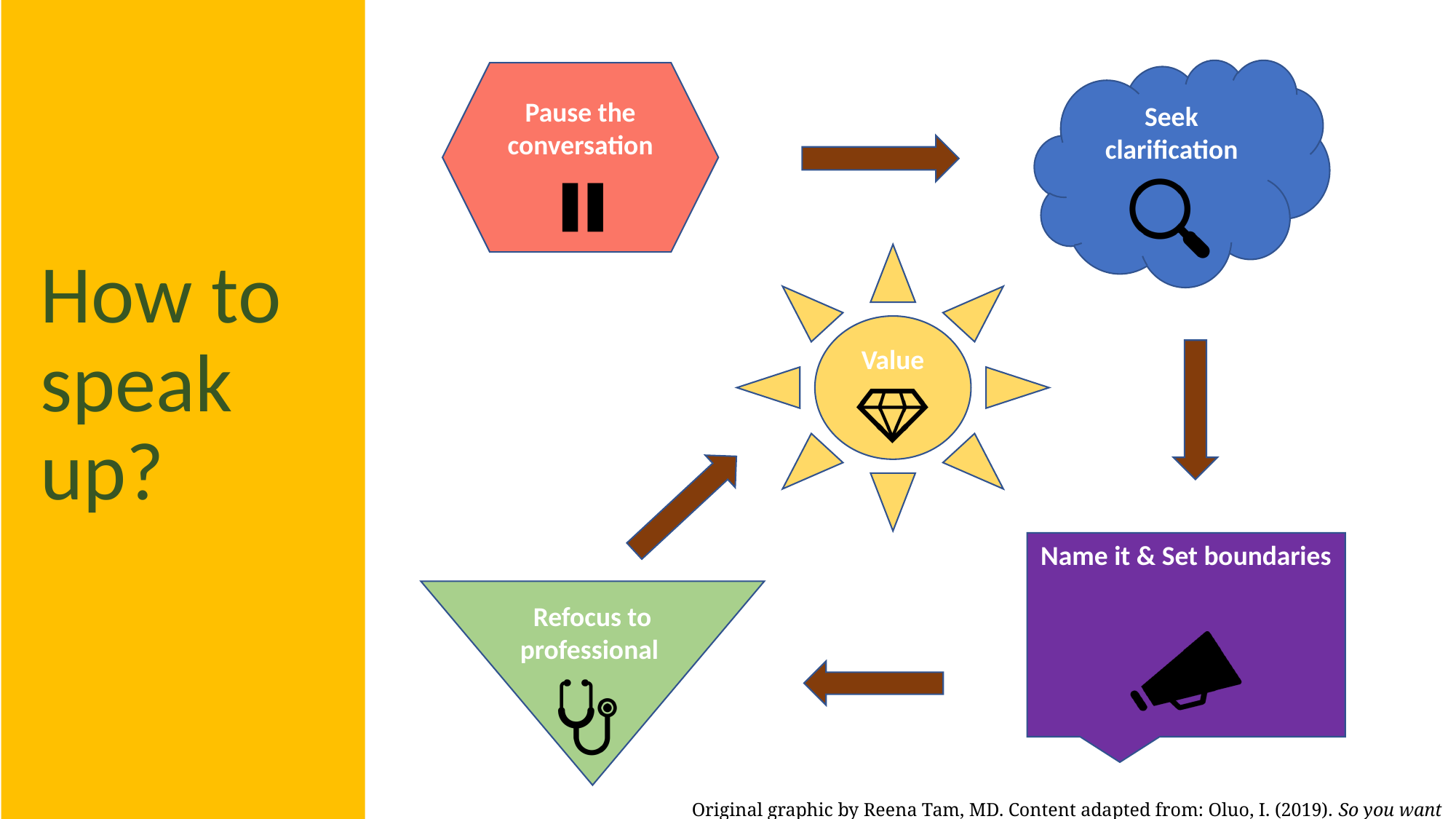

How to speak up?
Seek clarification
Pause the conversation
Value
Name it & Set boundaries
Refocus to professional
Original graphic by Reena Tam, MD. Content adapted from: Oluo, I. (2019). So you want to talk about race. Seal Press.

## Slide 17
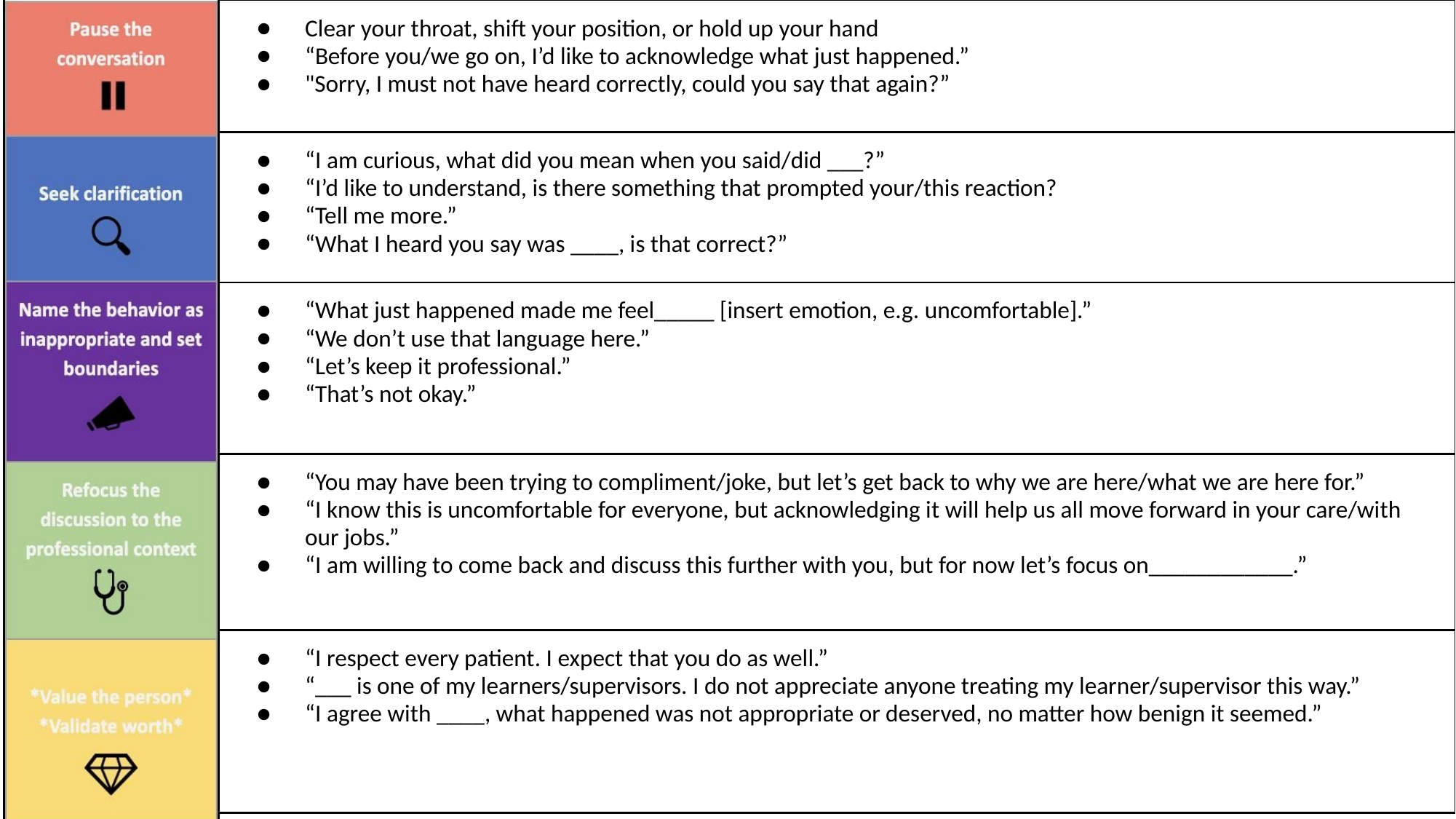

| Clear your throat, shift your position, or hold up your hand “Before you/we go on, I’d like to acknowledge what just happened.” "Sorry, I must not have heard correctly, could you say that again?” |
| --- |
| “I am curious, what did you mean when you said/did \_\_\_?” “I’d like to understand, is there something that prompted your/this reaction? “Tell me more.” “What I heard you say was \_\_\_\_, is that correct?” |
| “What just happened made me feel\_\_\_\_\_ [insert emotion, e.g. uncomfortable].” “We don’t use that language here.” “Let’s keep it professional.” “That’s not okay.” |
| “You may have been trying to compliment/joke, but let’s get back to why we are here/what we are here for.” “I know this is uncomfortable for everyone, but acknowledging it will help us all move forward in your care/with our jobs.” “I am willing to come back and discuss this further with you, but for now let’s focus on\_\_\_\_\_\_\_\_\_\_\_\_.” |
| “I respect every patient. I expect that you do as well.” “\_\_\_ is one of my learners/supervisors. I do not appreciate anyone treating my learner/supervisor this way.” “I agree with \_\_\_\_, what happened was not appropriate or deserved, no matter how benign it seemed.” |

## Slide 18
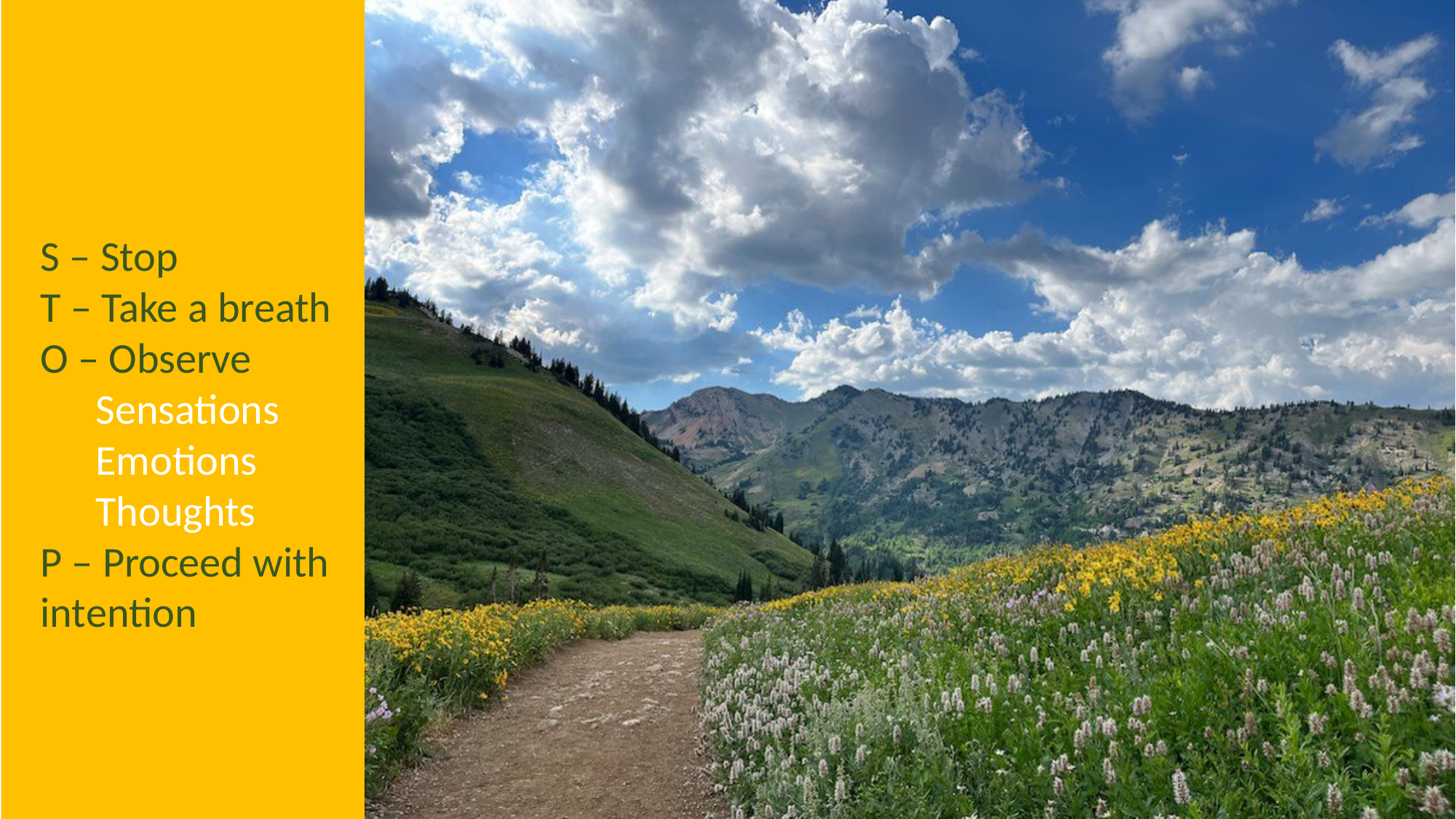

S – Stop
T – Take a breath
O – Observe
Sensations
Emotions
Thoughts
P – Proceed with intention

## Slide 19
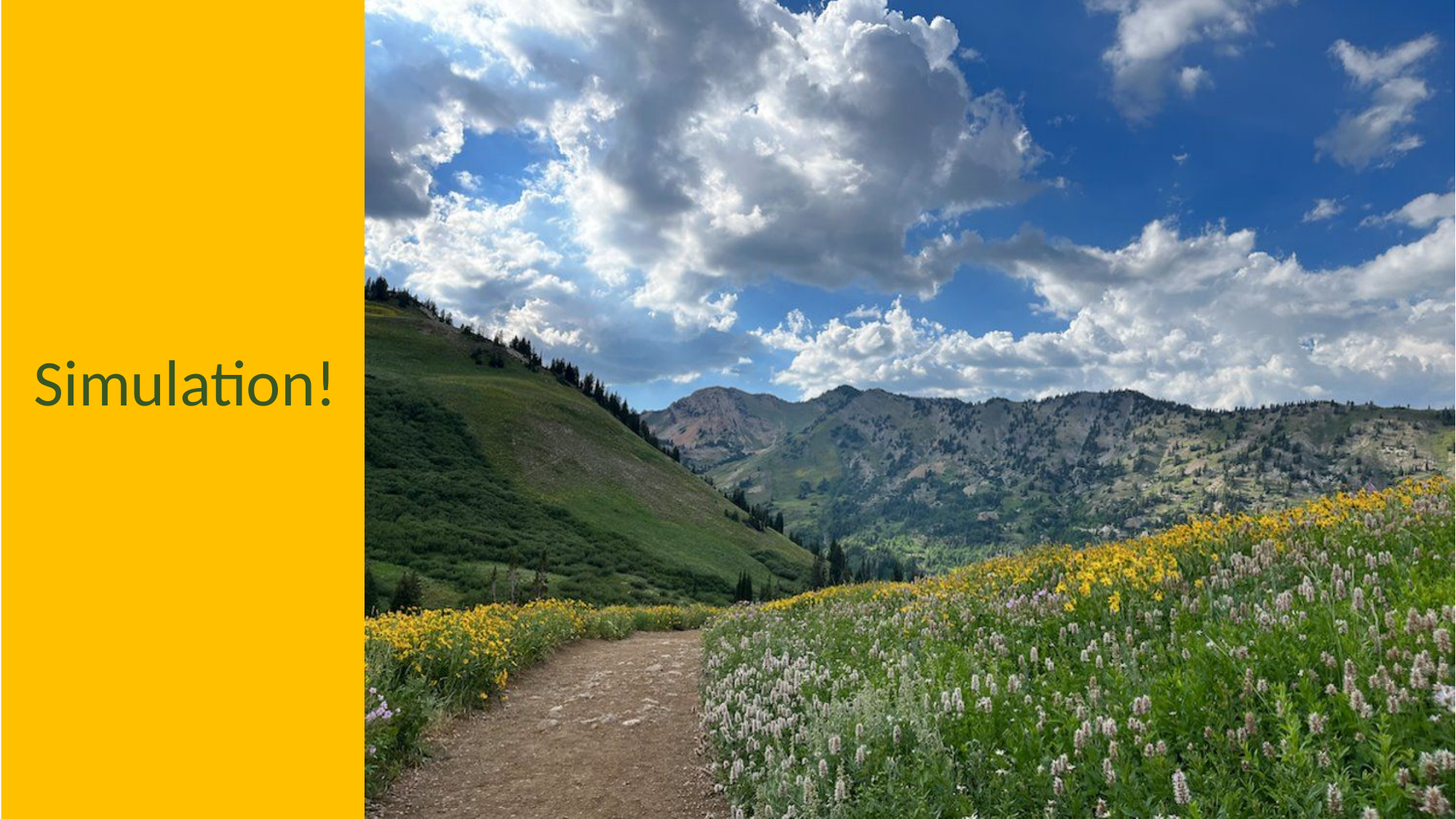

Simulation!

## Slide 20
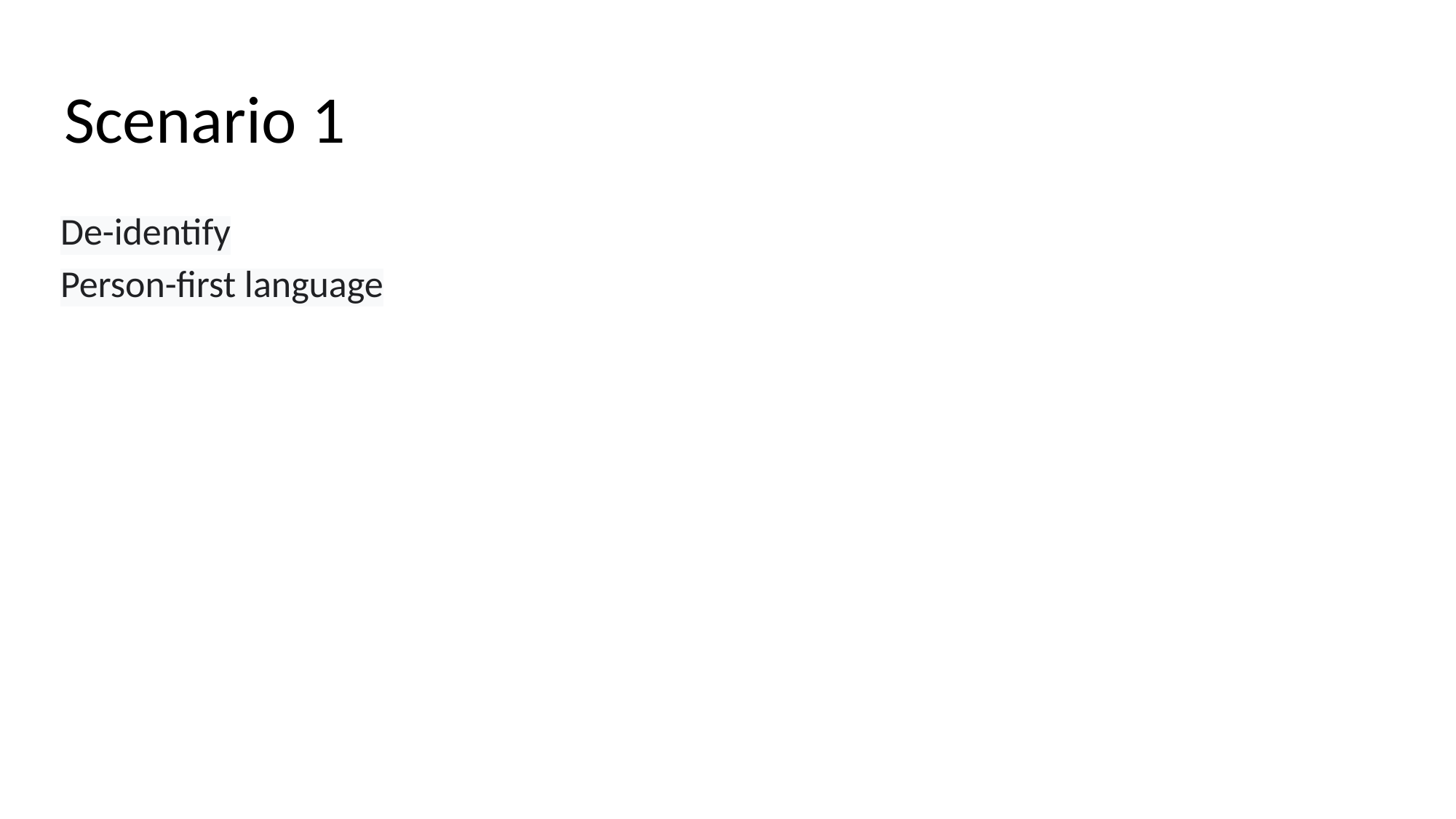

# Scenario 1
De-identify
Person-first language

## Slide 21
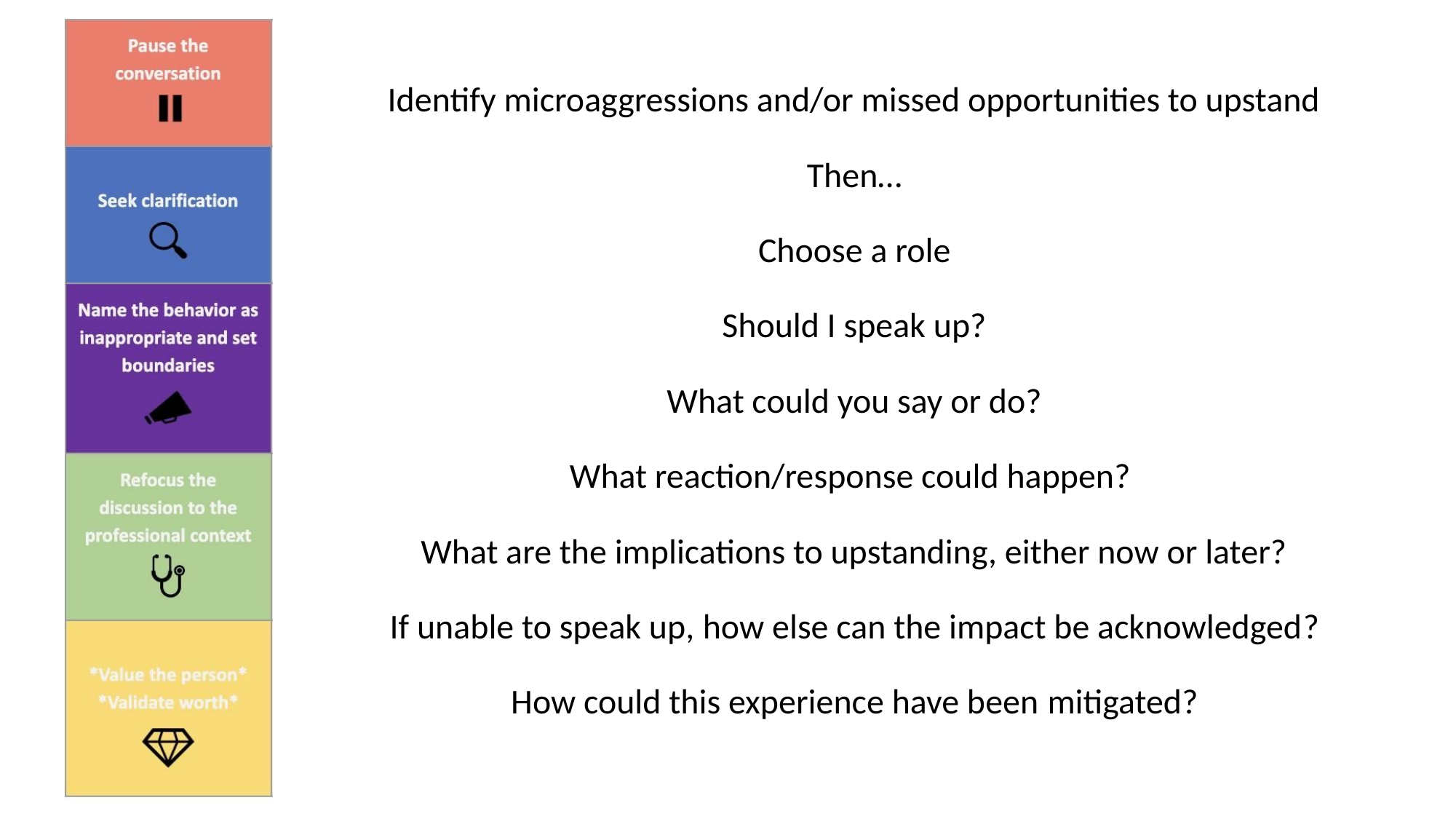

Identify microaggressions and/or missed opportunities to upstand
Then…​
Choose a role
Should I speak up?
What could you say or do?​
​
What reaction/response could happen? ​
​
What are the implications to upstanding, either now or later?​
If unable to speak up, how else can the impact be acknowledged?
​
How could this experience have been mitigated?​
​

## Slide 22
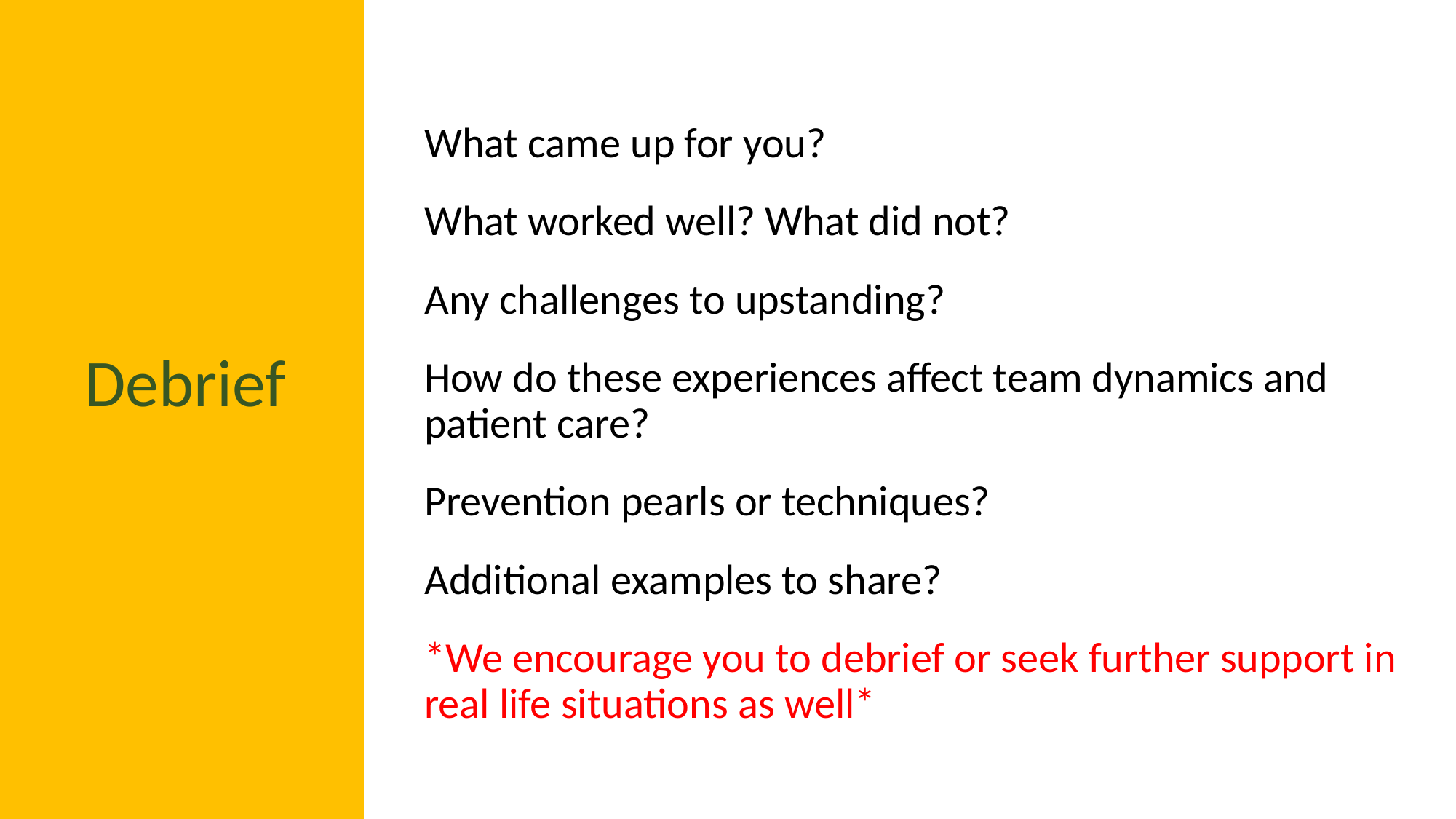

Debrief
What came up for you?
What worked well? What did not?
Any challenges to upstanding?
How do these experiences affect team dynamics and patient care?
Prevention pearls or techniques?
Additional examples to share?
*We encourage you to debrief or seek further support in real life situations as well*

## Slide 23
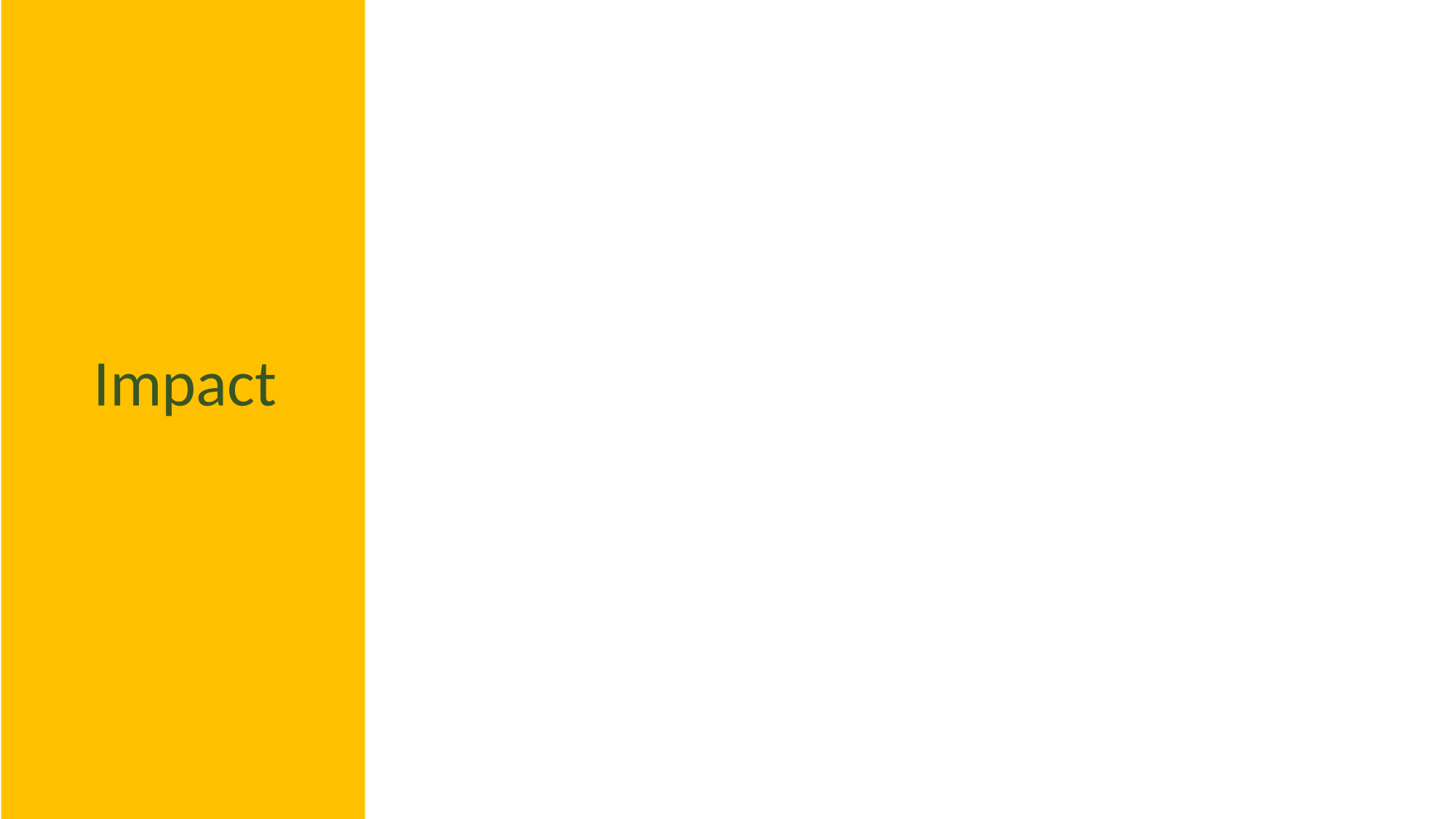

Impact

## Slide 24
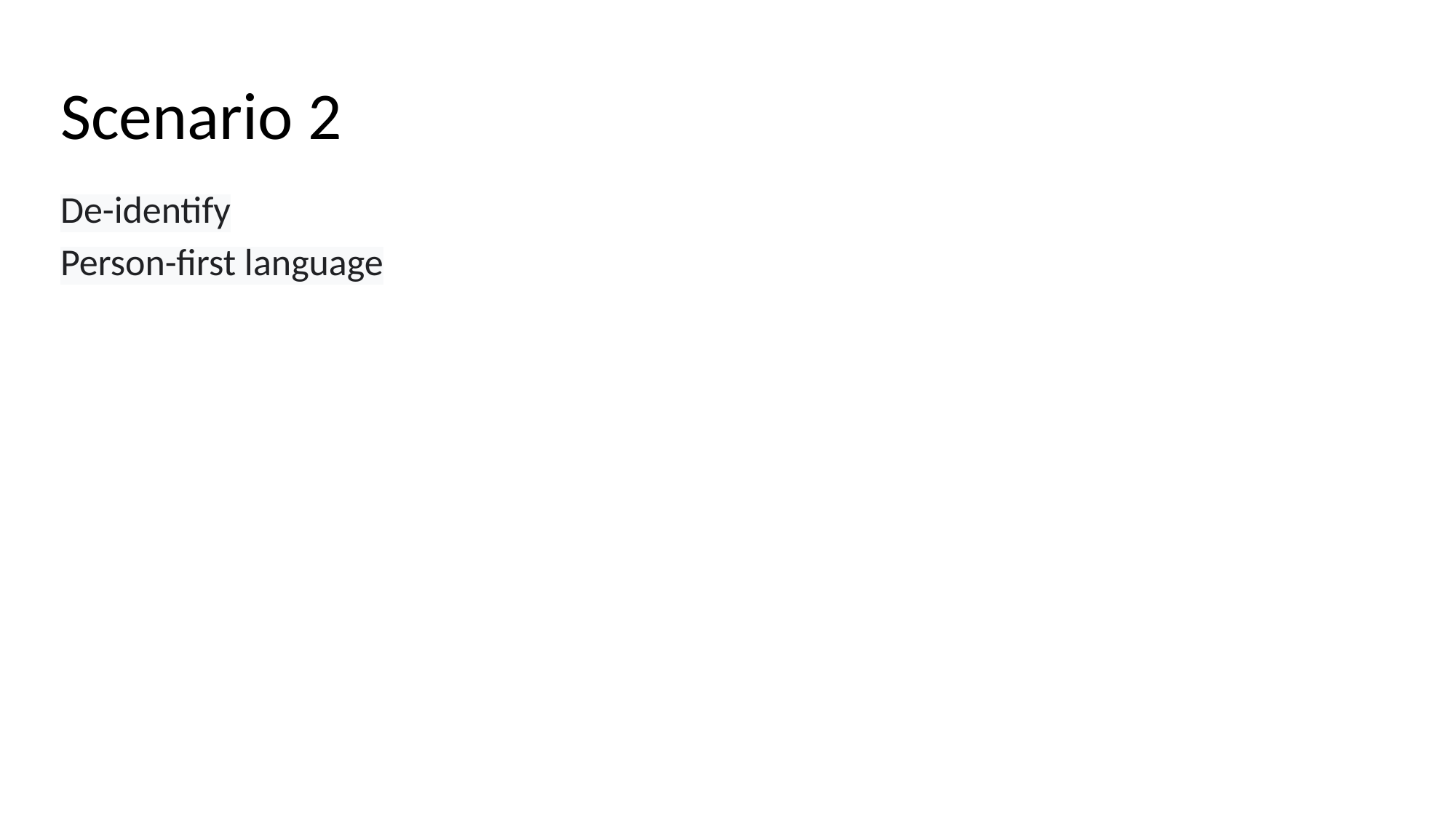

# Scenario 2
De-identify
Person-first language

## Slide 25
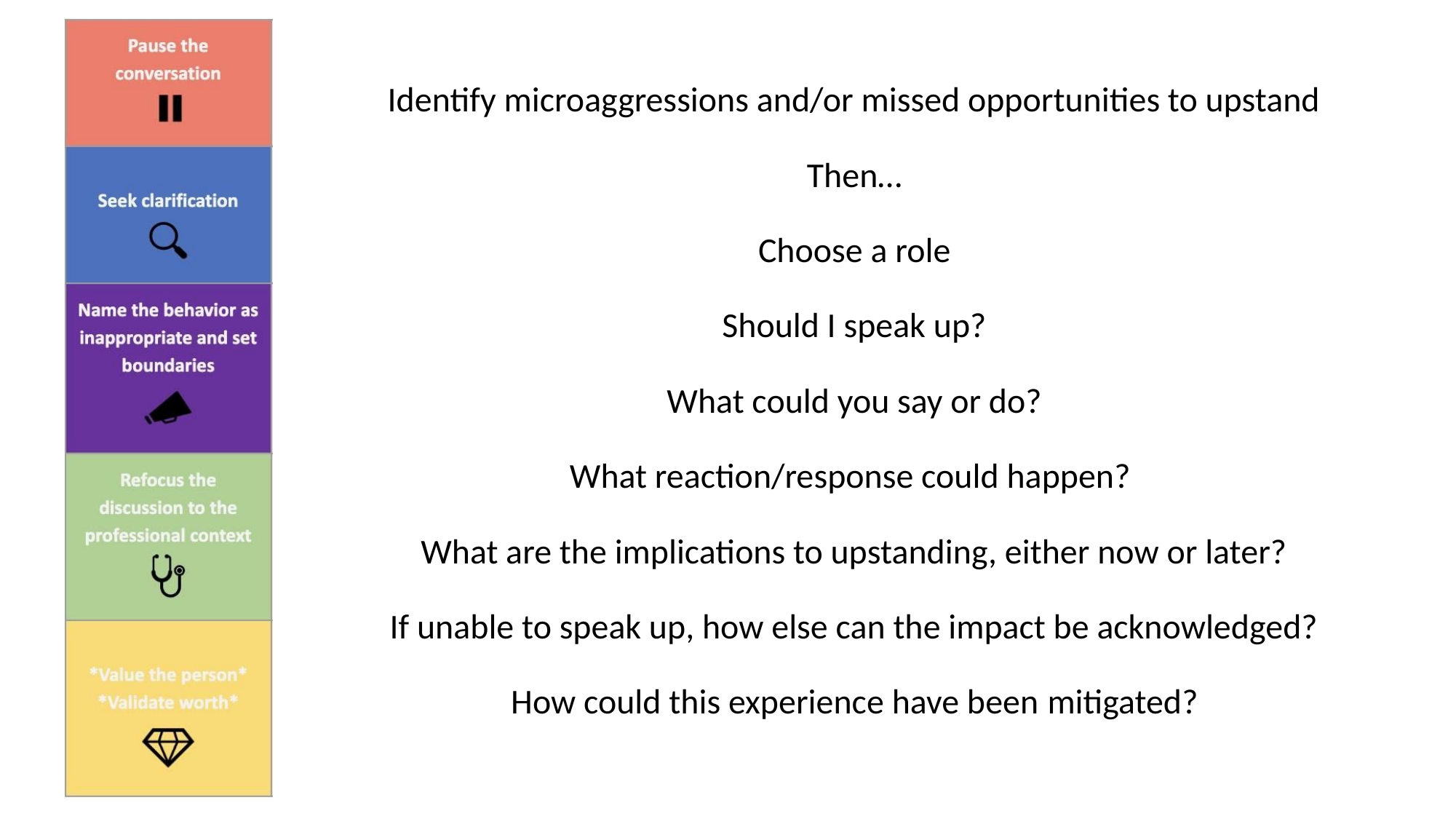

Identify microaggressions and/or missed opportunities to upstand
Then…​
Choose a role
Should I speak up?
What could you say or do?​
​
What reaction/response could happen? ​
​
What are the implications to upstanding, either now or later?​
If unable to speak up, how else can the impact be acknowledged?
​
How could this experience have been mitigated?​​

## Slide 26
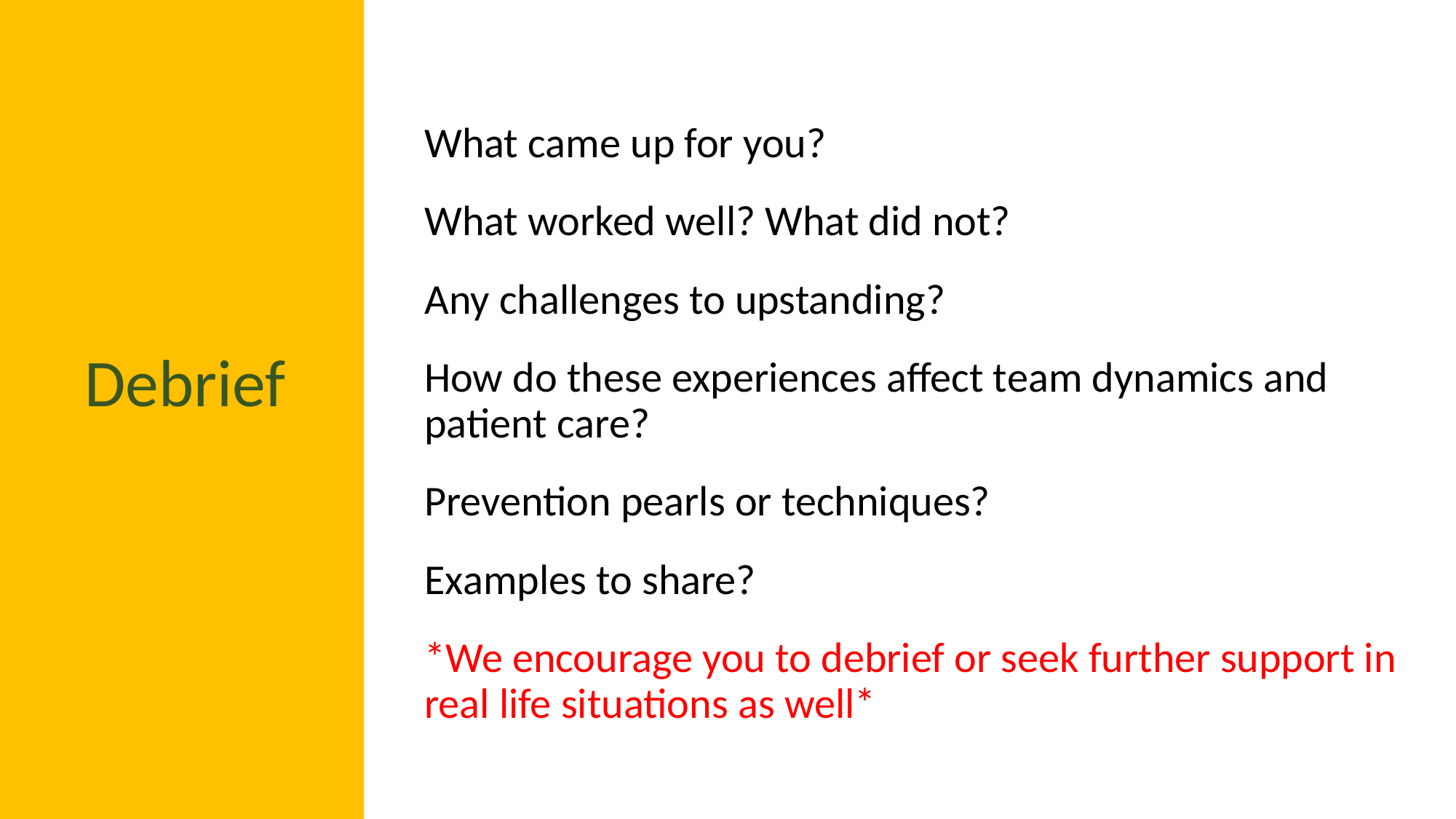

Debrief
What came up for you?
What worked well? What did not?
Any challenges to upstanding?
How do these experiences affect team dynamics and patient care?
Prevention pearls or techniques?
Examples to share?
*We encourage you to debrief or seek further support in real life situations as well*

## Slide 27
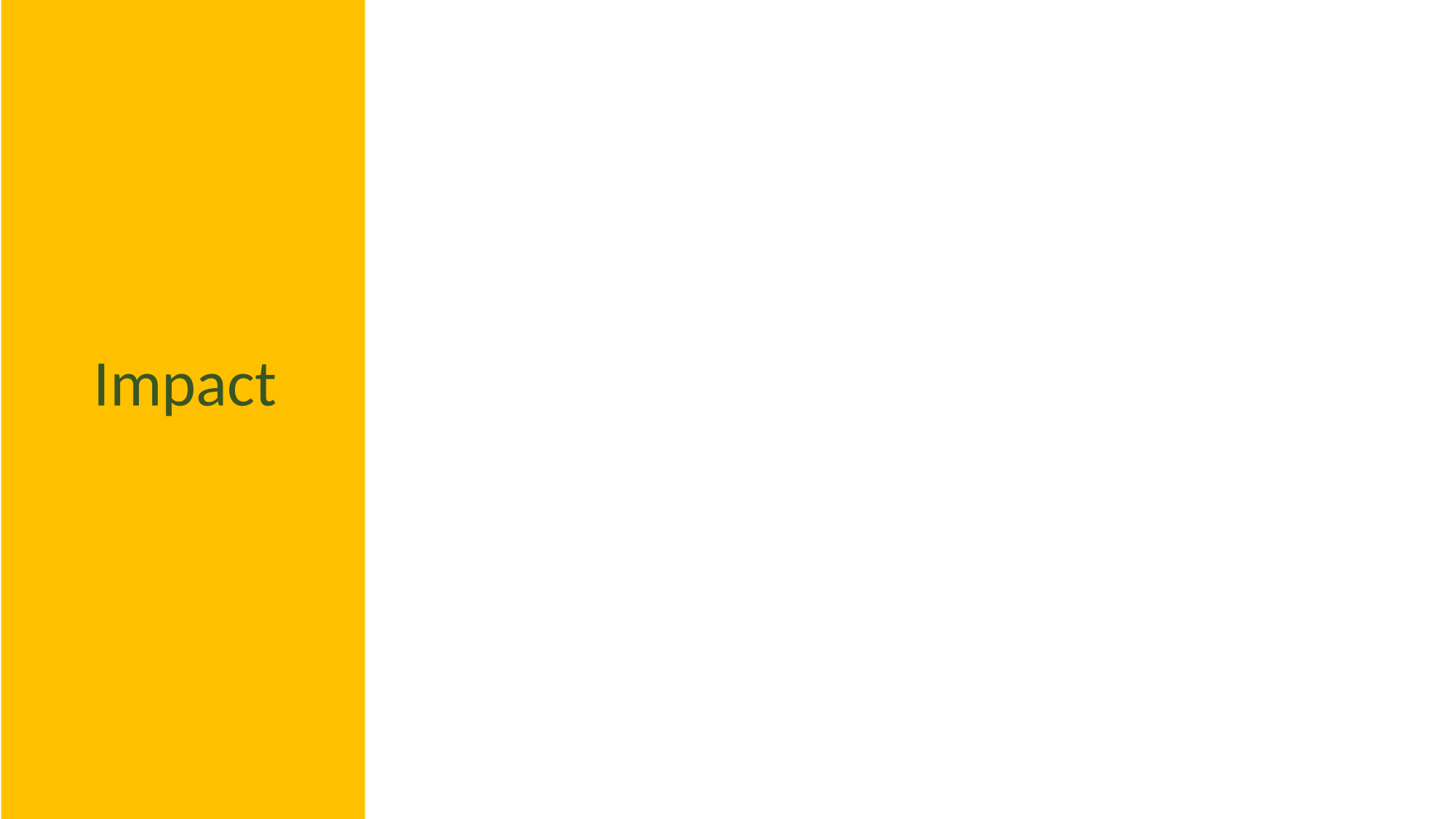

Impact

## Slide 28
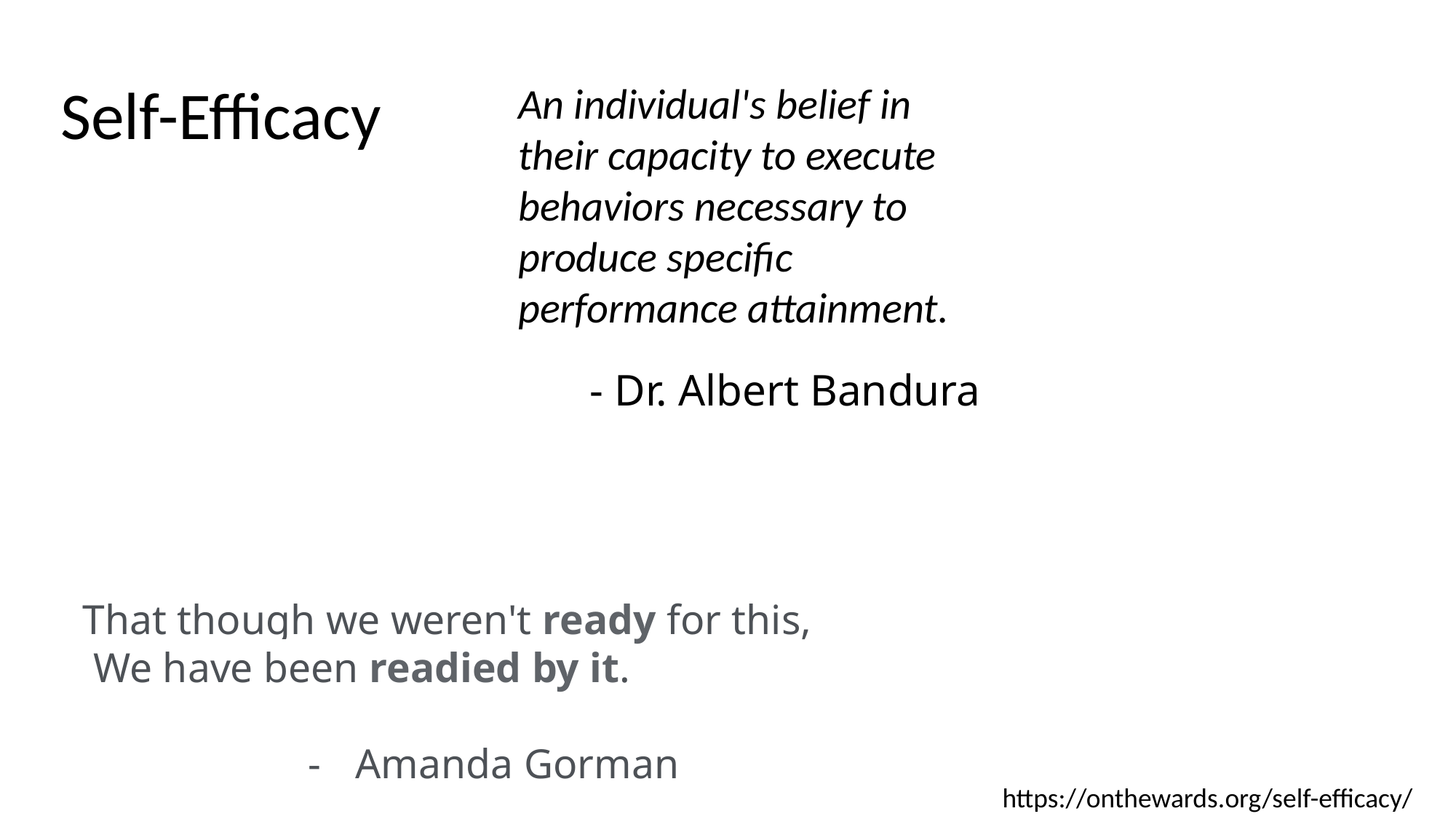

# Self-Efficacy
An individual's belief in their capacity to execute behaviors necessary to produce specific performance attainment.
- Dr. Albert Bandura
That though we weren't ready for this,
 We have been readied by it.
Amanda Gorman
https://onthewards.org/self-efficacy/

## Slide 29
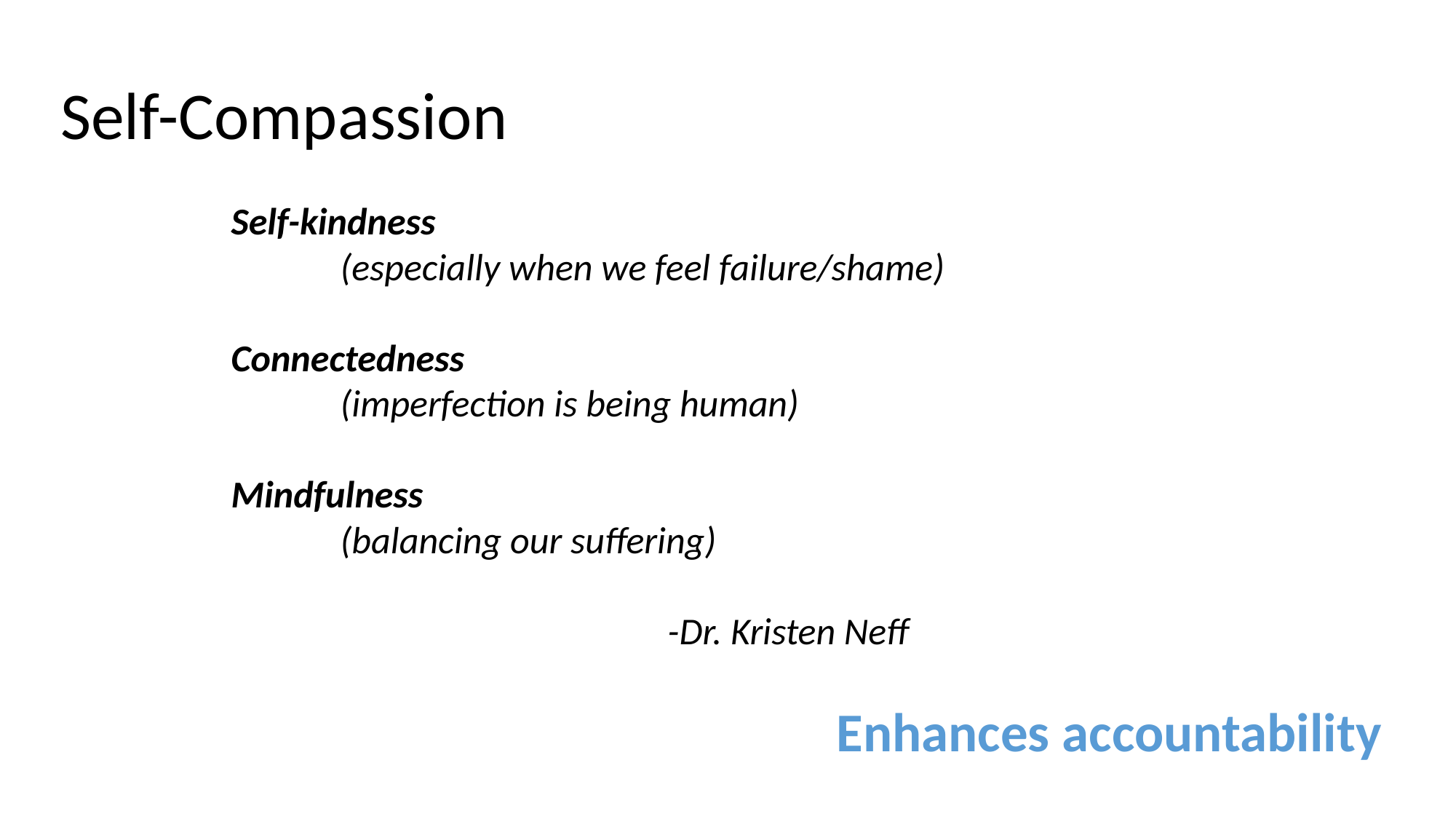

# Self-Compassion
Self-kindness
	(especially when we feel failure/shame)
Connectedness
	(imperfection is being human)
Mindfulness
	(balancing our suffering)
				-Dr. Kristen Neff
Enhances accountability

## Slide 30
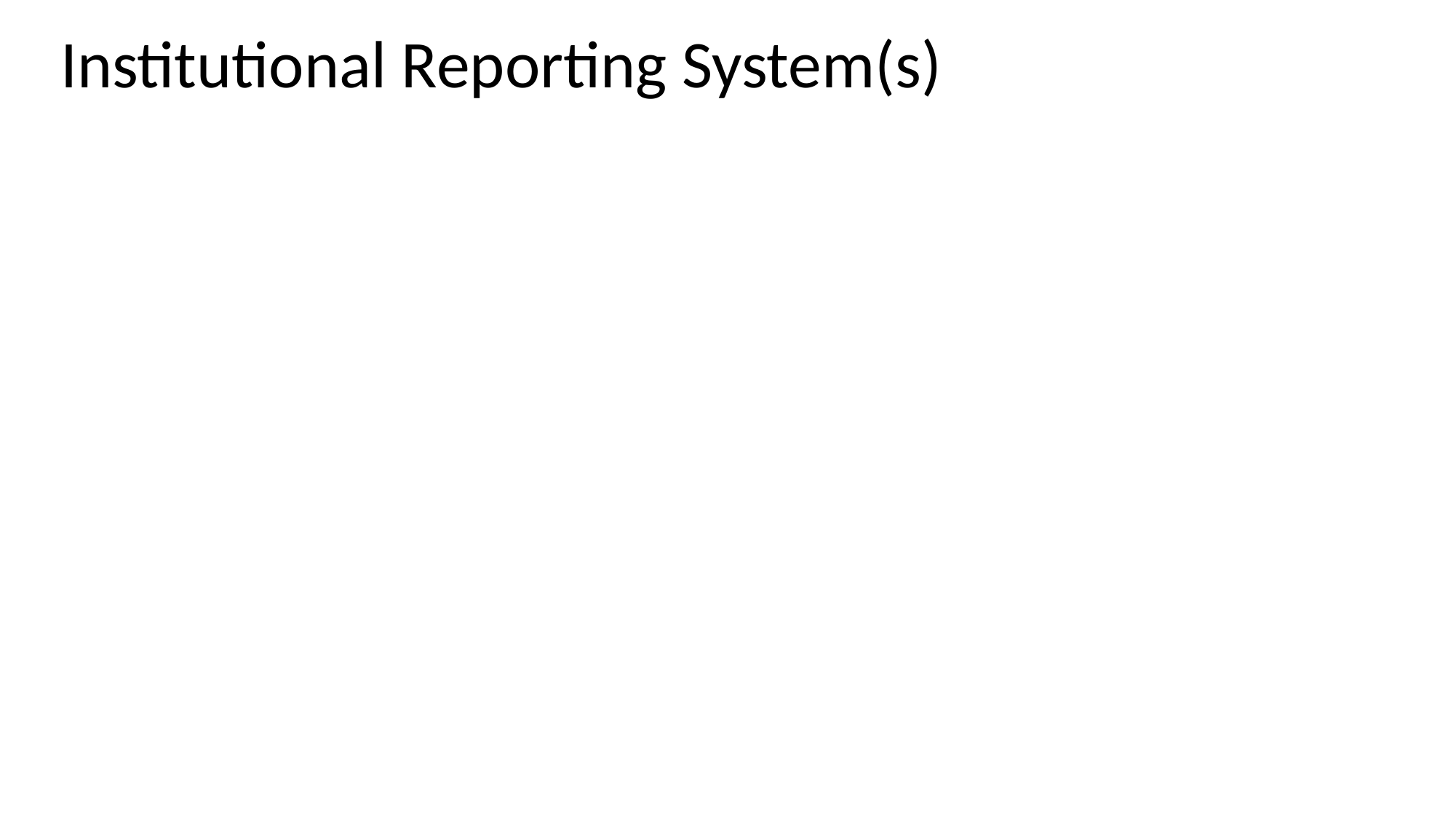

# Institutional Reporting System(s)

## Slide 31
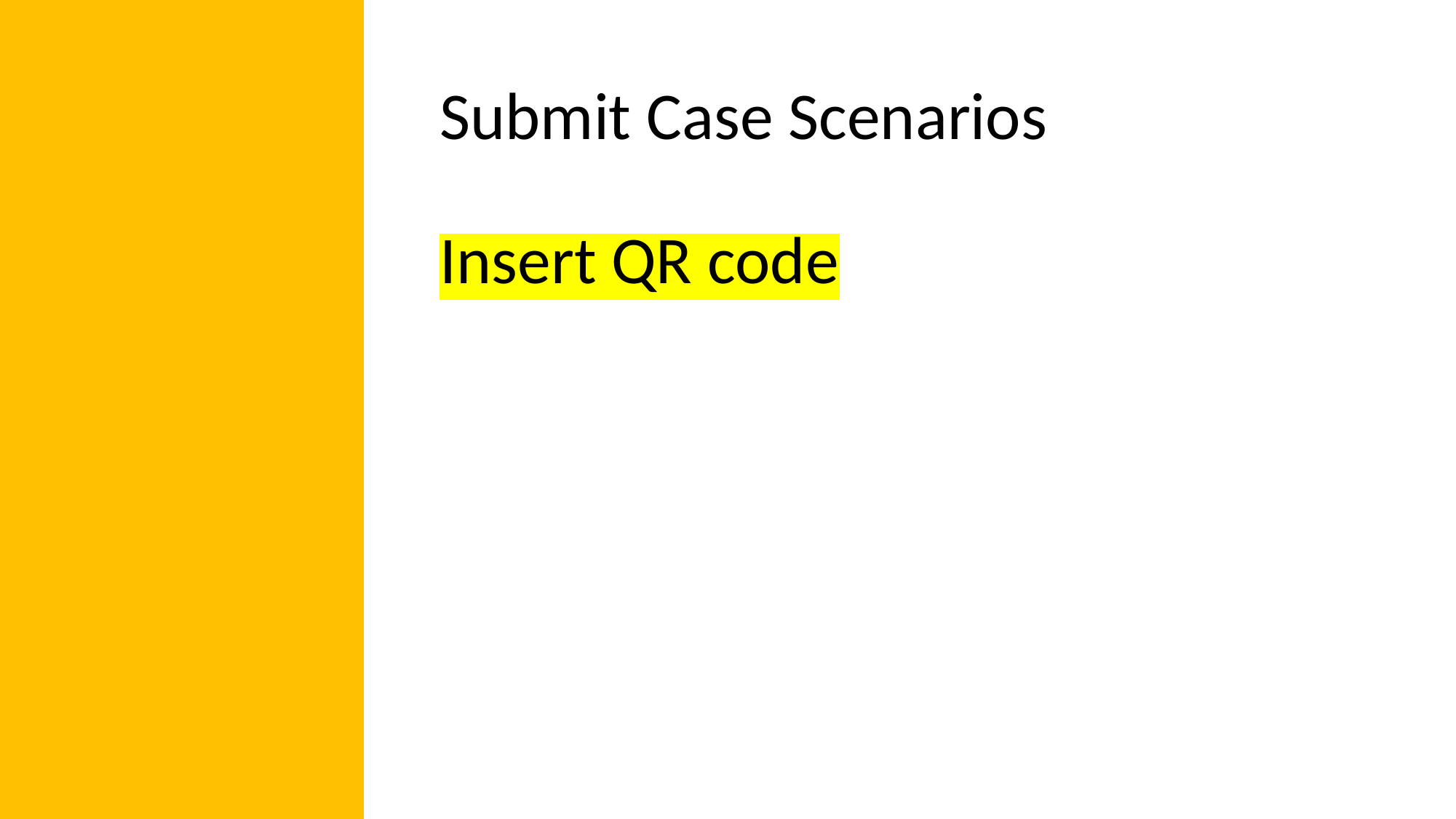

# Submit Case Scenarios
Insert QR code

## Slide 32
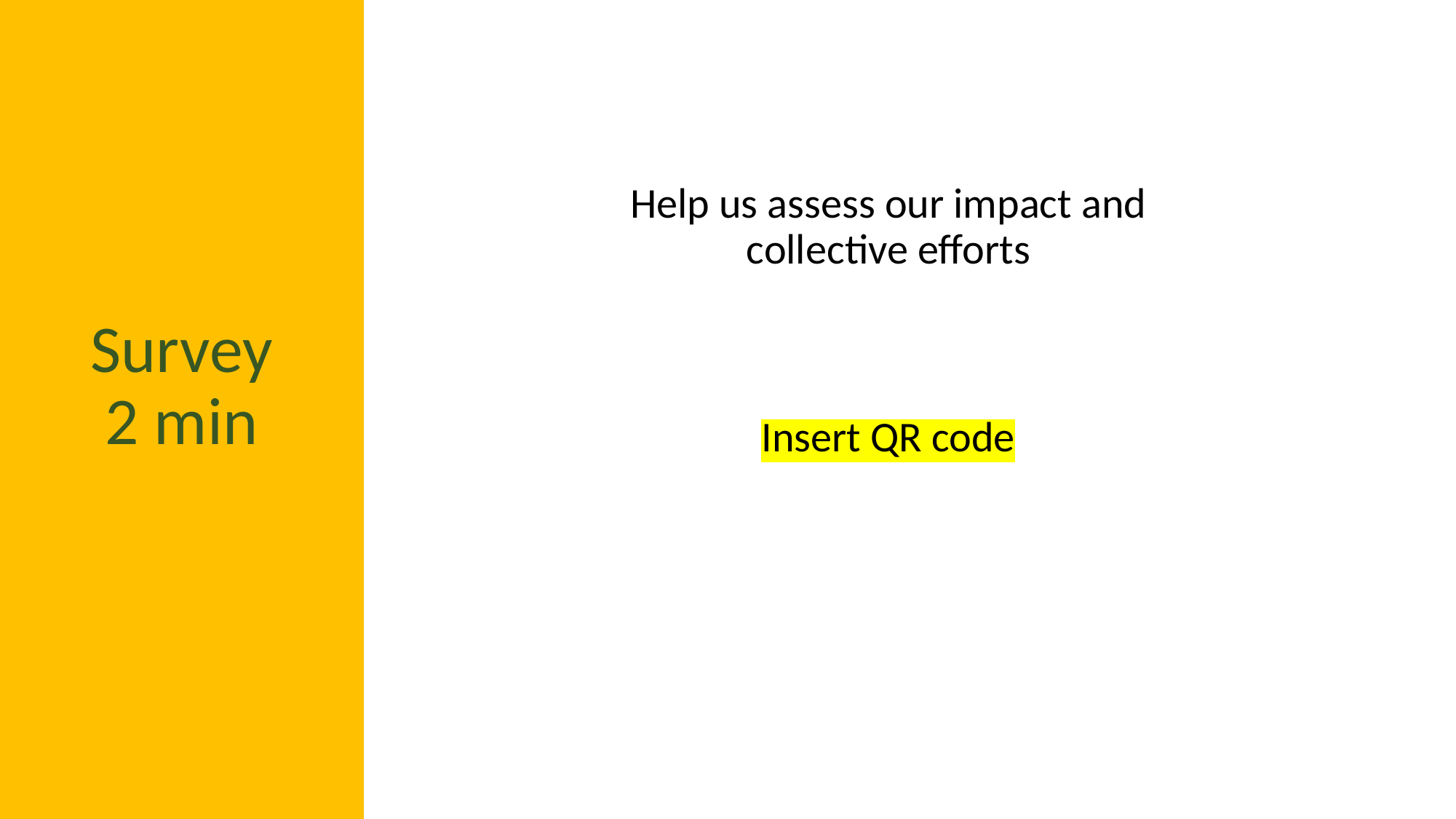

Survey
2 min
Help us assess our impact and collective efforts
Insert QR code
