# Supplementary material for: Speak Up! Simulation Workshop: Teaching Graduate Medical Trainees to Recognize and Respond to Microaggressions in the Clinical Setting
Source: MedEdPORTAL. 2025 Aug 29;21:11545. doi: 10.15766/mep_2374-8265.11545 (PMC12394545; doi:10.15766/mep_2374-8265.11545)
Supplement: Supplementary file 1 — Speak Up! Simulation Workshop - Template.pptxFacilitator Guide and Agenda.docxPostworkshop Survey.docxParticipant Speak Up! Guide.docxDeidentified Microaggression Case Bank.pptx [file mep_2374-8265.11545-s001.zip › B. Facilitator Guide and Agenda.docx]

Instructional Use

**Schedule for the workshop:**

- The original Speak Up! Simulation workshops were conducted over a 90-minute period, however, it can be adaptable to your institution's scheduling capacity and interest. Extended sessions such as half or full day workshops or divided sessions over multiple days, could be achieved by giving more time for small group and large group discussions, integrating additional cases, including more didactic time reviewing published literature, demonstrating how to access institutional reporting structures or submitting their own case scenario, and lastly, providing time to administer separate pre- and post-surveys of desired, rather than one post-workshop survey. If dividing the workshop, ensure adequate time is spent on psychological safety, and review definitions and the framework before role play each day.
- Use this guide to prepare for each workshop

**Preparing this guide:**

- Update the sample schedule below
- Develop and insert case-specific text below (red font below)
- Print and/or email this facilitator guide

Facilitator Guide and Agenda

(*distribute or print for facilitation use*)

Required time: 90 minutes

Materials needed: laptop, adapter, projector, printed participant guide, easel/marker (optional)

Date:

Time:

Location:

Facilitators:

Schedule:

| **Time \| Length** |  |
| --- | --- |
| 00:00 - 5-7 min | Introductions |
| 00:00 - 7 min | Psychological Safety |
| 00:00 - 3 min | Purpose/Intention |
| 00:00 - 12 min | Definitions |
| 00:00 - 2 min | When to Speak Up |
| 00:00 - 5 min | How to speak up |
| 00:00 - 5 min | STOP meditation exercise and/or BREAK |
| 00:00 - 10-15 min | Scenario #1 - small group |
| 00:00 - 3-5 min | Debrief and Impact |
| 00:00 - 10-15 min | Scenario #2 - small group |
| 00:00 - 3-5 min | Debrief and Impact |
| 00:00 - 3 min | Self-Compassion, Self-Efficacy |
| 00:00 - 3 min | Reporting and Submitting Scenarios |
| 00:00 - 2-3 min | Survey |
| = 90 minutes | Adjourn |

**Running the workshop:**

- Ensure technology is working including screen sharing.
- Start on time as much as possible - the time goes by fast
- Preparing for small groups role play:
  - In-person only: count off participants based on number of facilitators or divide the audience into even sections, then move to dedicated space facing each other.
  - Virtual only: divide participants into breakout rooms based on number of facilitators
  - Hybrid: ensure adequate number of facilitators are present in each space. Divide participants, same as above.

**Facilitating small groups:** (repeat for each scenario)

**Scenario:** [Insert case script] - optional

- Read off case scenario to small group or allow for them to read in silence
- Ask participants to identify microaggressions
- Ask participants to self-select roles
- Use guiding questions below

*Guiding questions:* [insert sample answers to each question]

- Should I speak up?
- What could you say or do?
- What reaction/response could happen? ​
- What are the implications to upstanding, either now or later?​
- If unable to speak up, how else can the impact be acknowledged?
- How could this experience have been mitigated?​
  ​

*Should I speak up? -* Weigh the risks and benefits:

1. If I respond, could my physical safety be in danger?

2. If I respond, will the person become defensive and will this lead to an argument?

3. If I respond, how will this affect my relationship with this person?

4. If I don’t respond, will I regret not saying something?

5. If I don't respond, does that convey that I accept the behavior or statement?

*How to speak up?* [use framework - insert appropriate talking points to each bullet point]

- Pause the conversation
- Seek clarification
- Naming behavior as inappropriate and setting boundaries.
- Refocus the discussion to the professional context.
- Value the person. Validate their worth.

*Additional teaching points:* [insert literature excerpts or statistics below]

- *Go deeper – gauge psychological safety first .* [insert “what if” challenge questions]

**Preparing for large group:**

- Provide a warning chime, bell, or alarm to bring the groups back in 1-2 minutes
- Close breakouts if virtual/hybrid, turn or bring chairs back to center
- Ask groups to share experiences using guiding questions

*Guiding questions - Debrief*

- What came up for you?
- What worked well? What did not?
- Any challenges to upstanding?
- How do these microaggressions affect team dynamics and patient care?
- Examples to share?
- *Encourage your participants to seek further support in real life situations as well*

*Impact*

- Highlight main points from the published literature you choose to include
- Invite questions from the audience

**Ending the workshop:**

- Be mindful of time - this fosters psychological safety
- Ensure enough time is left for survey completion, if applicable
- Relay availability for further debriefing or discussion, if desired
