# Supplementary material for: Speak Up! Simulation Workshop: Teaching Graduate Medical Trainees to Recognize and Respond to Microaggressions in the Clinical Setting
Source: MedEdPORTAL. 2025 Aug 29;21:11545. doi: 10.15766/mep_2374-8265.11545 (PMC12394545; doi:10.15766/mep_2374-8265.11545)
Supplement: Supplementary file 1 — Speak Up! Simulation Workshop - Template.pptxFacilitator Guide and Agenda.docxPostworkshop Survey.docxParticipant Speak Up! Guide.docxDeidentified Microaggression Case Bank.pptx [file mep_2374-8265.11545-s001.zip › _Educational Summary Report_11545.pdf]

MedEdPORTAL® The AAMC Journal of Teaching and Learning Resources

Original Publication Open Access

Speak Up! Simulation Workshop: Teaching Graduate Medical Trainees to Recognize and Respond to Microaggressions in the Clinical Setting

Nathaniel E. Hayward, MD, MSc*, Amanda J. McLearn-Montz, MD, Katherine Amano, MD, MA, Quang-Tuyen Nguyen, MD, Rebecca Purtell, MD, Katie Gradick, MD, MHS†, Reena P. Tam, MD†

*Corresponding author: nathaniel.hayward@gmail.com
†Co-senior author

Abstract

Introduction: The negative effects of microaggressions on patient care, provider well-being, and medical education are well-documented. Critically evaluated programs addressing microaggressions remain largely absent in the literature. **Methods:** We developed the 90-minute Speak Up! Simulation Workshop for GME trainees (residents, fellows), physician attendings, other health care professionals (e.g., nurses, nurse practitioners, child life specialists) based on Kolb's Experiential Learning Cycle. We utilized deidentified cases reported at our institution, emphasizing psychological safety and upstanding. Based on Kirkpatrick's Evaluation Model, we distributed pre- and postworkshop surveys to assess perceptions of psychological safety, confidence in recognizing microaggressions, comfort in upstanding, and likelihood of addressing future discrimination incidents. Five-point Likert scales and Bowker's symmetry tests were used. **Results:** Eighty anonymous postworkshop surveys were collected from GME trainees ($N = 151$) at 14 sessions. Of the respondents, 63 (79% [95% CI 70% to 88%]) reported feeling psychologically safe. When the 80 respondents compared their comfort in addressing microaggressions before and after the workshop, a significant shift was seen in the likelihood of speaking up when witnessing a microaggression (χ^2[df5] = 42, $p < .01$), with scores from 42 respondents (52%) increasing by at least 1 point. Significant shifts in confidence in identifying microaggressions also occurred (χ^2[df5] = 43, $p < .01$), with 43 respondents (54%) showing increased confidence. **Discussion:** Evaluation of our curriculum demonstrated improved understanding of microaggressions and enhanced self-efficacy in upstanding. This simulation workshop provides psychologically safe opportunities to explore the impact of microaggressions and empowers participants to respond effectively in professional settings.

Keywords
Simulation, Psychological Safety, Microaggressions, Bias, Discrimination, Advocacy, Communication Skills, Diversity, Equity, Inclusion, Health Equity

Educational Objectives

By the end of this simulation workshop, learners will be able to:

1. Recognize implicit biases and microaggressions in a clinical setting.
2. Respond to deidentified microaggressions reported at their institution using a flexible framework.
3. Apply the concepts of psychological safety and upstanding to the clinical setting.
4. Examine personal and shared experiences of microaggressions and upstanding in the clinical setting.

Citation:
Hayward NE, McLearn-Montz AJ, Amano K, et al. Speak up! Simulation workshop: teaching graduate medical trainees to recognize and respond to microaggressions in the clinical setting. *MedEdPORTAL*. 2025;21:11545.
https://doi.org/10.15766/mep_2374-8265.11545

Introduction

The concept of implicit, unconscious bias posits that past experiences influence our judgment and actions, often without our awareness.[1] Implicit biases become problematic when they lead to discriminatory actions, including microaggressions. First defined in 1970, microaggressions are now understood as commonplace verbal or behavioral indignities, whether intentional or unintentional.[2,3]

Microaggressions have harmful implications for both patients and health care professionals in clinical and medical education settings. Patients who experience microaggressions have poorer outcomes, including increased rates of depression

Copyright © 2025 Hayward et al. This is an open-access publication distributed under the terms of the Creative Commons Attribution license.

and medical mistrust.[4] Similarly, health care trainees who experience microaggressions report higher levels of burnout and depression.[5,6] These negative effects are amplified when learners feel helpless to respond or question the validity of their experience.[3] To address these issues, we developed the Speak Up! Simulation Workshop to equip GME trainees with the knowledge and practical skills needed to respond to microaggressions in the role of upstanders who can actively address these dilemmas within our institution. In this context, upstanding refers to promoting positive social change by taking a stand against injustice, bullying, intolerance, or harm.

While this workshop with small-group simulations is relevant for all health care professionals, we targeted GME trainees. These individuals are more likely than faculty members to witness and experience microaggressions, due to the hierarchical nature of medical training and the vulnerability inherent as a learner.[7] Although national data on mistreatment of all GME trainees are lacking, two studies have reported high rates of mistreatment among surgical residents. From a sample of 7,409 residents, nearly 50% reported experiencing some form of mistreatment (based on gender, race, or other social identities), with 65% of female residents reporting gender discrimination and 20% reporting sexual harassment.[8] A 2020 institutional survey found similar rates, with 48% of residents and 29% of clinical faculty reporting experiencing mistreatment.[9]

Our simulation builds on similar published curricula, including one study that also focused on cultivating and evaluating psychological safety,[10] which is defined as a shared belief that participants can engage in interpersonal risk taking without shaming or humiliation.[11] For our simulation, we utilized cases from a continuously updated pool of deidentified microaggressions witnessed or experienced at our institution. Additionally, whereas some publications have focused on equipping learners with the ability to address microaggressions from patients,[12-25] our cases also included instances in which trainees practice responding to microaggressions perpetuated by other members of the health care team. Most studies of comparable workshops, including one that evaluated psychological safety,[10] involved a smaller total number of participants,[14] and studies that involved larger sample sizes[12,13] did not explicitly assess psychological safety. Our intervention includes an assessment of trainee psychological safety. The Speak Up! Simulation Workshop has been implemented a total of 27 times with more than 700 participants to date, including 15 workshops specifically for GME trainees, and has been refined over 4 years.

Microaggressions often stem from power disparities; thus, learning to respond in situations where trainees face power differentials (e.g., attending physician acting as an aggressor toward a resident) is critical for fostering a more equitable culture in medical education. Our intervention aims to provide a unique framework for individual and collective responses, while allowing for nuance and flexibility in group learning. Additionally, our curriculum covers a diverse range of microaggressions, including discrimination based on language preference, an area often overlooked but well documented as a source of inequity in health care.[26,27]

Methods

Design

The curriculum was developed by a team of faculty and trainees, including the authors, who were motivated to respond to gaps identified in GME curricula, local needs assessment informed by institutional safety data, and the lived experiences from our facilitators who had experienced or witnessed microaggressions in our institution. This team of faculty and trainees who developed the curriculum had previous educational, leadership, and research experience in equity work. Trainee involvement in facilitation was vital to ensuring that simulation cases were relevant to trainee lived experience and enhanced content accessibility and psychological safety in the workshop.

The curriculum's design was informed by Kolb's Experiential Learning Cycle and used a flexible framework adapted from that described by Ijeoma Oluo in *So You Want to Talk About Race*,[28] coupled with palliative care tools for teaching difficult conversations.[29] Trainees participated in small-group discussions in which they role-played and practiced strategies, such as pausing the conversation, seeking clarification, naming the behavior, refocusing on professional context, and acknowledging the inherent value of those targeted by microaggression.[28] Given the psychological distress that can accompany these conversations, even in simulation, palliative communication tools were employed as facilitation techniques known to be effective for teaching trainees to navigate difficult conversations, including the option to pause a simulation to seek collaboration and support if a participant is struggling.[29] Small-group discussions are intended to emphasize the nuanced application of these strategies in various scenarios, considering roles such as witness, aggressor, or victim, and recognizing the power dynamics typical of medicine. Avoiding retraumatization is a core component of psychological safety, and in an ideal setting, standardized actors would be used to avoid any risk of retraumatization.[10] If funding is not available for standardized actors, we believe this workshop

Copyright © 2025 Hayward et al. This is an open-access publication distributed under the terms of the Creative Commons Attribution license.

could still be effectively used if facilitators are explicit in their introductory discussion that no role is obligatory, that no one is asked to play a role because of an identity they hold, that only facilitators play the role of victims and/or aggressors, and that we only ask participants to play provider roles within the health care system (e.g., no one is asked to represent a patient). At the beginning of each session, we emphasize that any participant can opt out and remove themselves from the simulation or workshop entirely, if they feel that is needed for their own self-care.

The target audience for these workshops includes GME trainees (medical residents and fellows), attending faculty physicians, and other health care professionals (e.g., nurses, nurse practitioners, child life specialists). In this report, we analyze the feedback obtained from a cohort of 80 GME trainees.

Facilitator Development
This workshop was conducted within existing educational venues for GME programs at the University of Utah, as well as through sessions for groups of health care professionals at Primary Children's Hospital in Utah. Facilitators were expected to have contributed to the workshop content development and to have previously participated in a microaggression simulation. Prior to the workshops, facilitators were required to familiarize themselves with facilitation techniques, our flexible upstanding framework, and the facilitators' guide specific to each case simulation (Appendices A and B). Experienced facilitators led mindfulness exercises to create a psychologically safe space prior to simulations and associated discussion.

Equipment/Environment
A standardized PowerPoint presentation was used for all workshops, which was modified by reviewing selected cases and evidence-based learning points after debriefing (Appendix A). For in-person sessions, a projector, computer, and space that allowed for small-group discussions were required. For virtual or hybrid sessions, the Zoom videoconferencing platform was used, with the breakout group feature dividing participants into discussion groups. Anonymous retrospective postworkshop surveys (Appendix C), and then preworkshop surveys, were created using Google Forms. Participants accessed the surveys using QR code hyperlinks on their phones during 10 minutes of protected time at the end of the session. The simulation workshops were optional offerings, held during established times for trainee didactics. We delivered content in alignment with the typical format of each educational venue (in-person, virtual, or hybrid).

Personnel
Each workshop required a minimum of two facilitators, whose primary role was to ensure psychological safety while helping navigate challenges arising during case discussion. An important portion of the introduction to the Speak Up! Simulation Workshop involved facilitators acknowledging their own social identities and the limitations imposed by those identities, and being explicit about past failures in upstanding to normalize the challenges involved. The effect of this was to model vulnerability and to reassure learners that they were not expected to bear the burden of teaching others, particularly if their lived experiences resonated with the simulated cases. Facilitators presented the didactic portion of each session, led small-group simulations and discussions, and managed the debriefing. We aimed to maintain a group size of between 10 and 15 individuals per facilitator to optimize interaction and opportunity for participation.

Implementation
The workshops were implemented in virtual, in-person, and hybrid formats, over 90 minutes (see Appendix B for example agenda). The structure of the workshop was based on Kolb's Experiential Learning Cycle conceptual framework of experiencing and reflecting.[30,31] The concrete experience (Kolb Stage 1) began with a brief didactic session to ensure a shared mental model of psychological safety, microaggressions, implicit bias, and upstanding.[27] Participants were then randomly divided into small groups to engage in the role-play–based simulation and subsequent case discussion (Appendix D). During simulation, participants volunteered to take on rotating bystander roles and were encouraged to imagine they were actively engaging in dialogue, though they could pause conversation at any time to ask for help from the group. Discussion prompts focused on identifying microaggressions and evaluating the associated risks and benefits faced by the individuals described in each case. Facilitators focused on strategies for approaching these challenges (Appendix B). Facilitators encouraged learners to pause the simulation and seek input from the group if they felt stuck. The large group reconvened for further discussion, allowing for reflection and observation (Kolb Stage 2) to occur.[31]

Debriefing
Following the discussions of the microaggressions, abstract conceptualization (Kolb Stage 3) of the learners' simulation experience was conducted via a structured debrief led by the facilitators.[31] The debrief portion served to share small-group discussion points and actionable skills that could be applied to real-world scenarios (Appendix E). The workshop concluded with key concepts to empower learners for active experimentation

Copyright © 2025 Hayward et al. This is an open-access publication distributed under the terms of the Creative Commons Attribution license.

(Kolb Stage 4),[31] such as the importance of self-compassion and self-efficacy when applying upstanding skills, mechanisms for reporting microaggressions and discrimination in clinical settings as patient safety events, obtaining mental health support, and completing voluntary surveys.

Data Analysis

Anonymous postworkshop surveys (Appendix C) were used to collect data on the effectiveness of the workshop and participant experiences, based on Kirkpatrick's validated Evaluation Model, which assesses reaction, learning, behavior, and outcomes; only the first two levels could be evaluated in our cohort without longitudinal follow-up.[32] The surveys assessed psychological safety, ability to recognize microaggressions, comfort responding to microaggressions, and likelihood of future upstanding in a clinical setting. The survey questions regarding level of confidence identifying microaggressions and level of comfort in likelihood of speaking up were assessed with 5-point Likert scales. Survey development was informed by a survey design workshop offered through our institution's Clinical and Translational Sciences Institute,[33] and the survey was reviewed by our department's medical education scholarship leaders. Data were anonymous, and therefore matched testing of postworkshop survey responses was not possible.

Pre- and postworkshop surveys were compared using Bowker's symmetry test, which is a generalization of the paired-sample McNemar test, for paired-sample crosstabulation tables larger than 2 x 2.[34] Responses to the free-text question "Did you feel psychologically safe during the session?" were consolidated using a screening code to distribute answers to nominal variables (responses of *Yes*, *Somewhat*, or *No*). Blank responses were labeled *Did not answer*.

Data were analyzed using Stata software (StataCorp, LLC). This study protocol received institutional review board approval by the University of Utah (IRB 00160216, deemed exempt December 22, 2022 and approved waiver of documentation of informed consent).

Results

After 14 workshops were conducted with 151 GME trainees, 80 postworkshop survey responses were received (53% response rate). In assessing psychological safety, 63 participants (79% [95% CI 70% to 88%]) indicated that they felt psychologically safe during the session (Table 1).

Table 1. Psychological Safety of GME Trainees During Speak Up! Simulation Workshop ($N = 80$)

Did You Feel Psychologically Safe During the Session?	Frequency (n)	Percent (%)
Yes	63	79
Somewhat	1	1
No	1	1
Did not answer	15	19
Total	80	100

Table 2 shows the distribution of Likert responses of participants who rated their pre- and postworkshop likelihood that they would speak up when witnessing microaggressions in a clinical setting. Bowker's test of symmetry for these responses ($N = 80$) showed that there was a highly significant shift from pre- to postworkshop in the likelihood that participants would speak up (χ^2[df5] = 42, $p < .01$). The majority of respondents ($n = 42$; 52%) experienced a change of at least 1 point on the Likert scale for their perceived comfort regarding likelihood of speaking up, while 47.5% of respondents ($n = 38$) did not experience a change in perceived likelihood following the simulation. No participants reported a decreased likelihood of speaking up following the simulation.

Table 3 shows the distribution of Likert responses of participants who rated their pre- and postworkshop confidence in identifying microaggressions in a clinical setting. Bowker's test of symmetry for these responses ($N = 80$) showed that there was a highly significant shift in participant confidence in identifying microaggressions from pre- to postworkshop (χ^2[df5] = 43, $p < .01$). The majority of respondents ($n = 43$; 53.8%) experienced a change of at least 1 point on the Likert scale in their perceived confidence level, while 47.5% of respondents ($n = 37$) did not experience any change in confidence. No participants reported decreased confidence in identifying microaggressions following the simulation.

Discussion

To address a gap in GME curricula related to recognizing and responding to microaggressions in the clinical setting, we developed a simulation workshop that aims to equip trainees with language and frameworks for upstanding. It features a palliative-care informed approach to simulation, utilizes

Table 2. Likert Responses for Pre- and Postworkshop Likelihood of Speaking Up When Witnessing a Microaggression in a Clinical Setting

Likelihood of Speaking Up[a]	Preworkshop n	Postworkshop n
Very likely	17	37
Likely	36	36
Unsure	19	6
Unlikely	6	1
Very unlikely	2	0
Total	80	80

[a]Participants rated their perceived likelihood on a 5-point Likert scale (1 = *very unlikely to speak up*, 5 = *very likely to speak up*).

Copyright © 2025 Hayward et al. This is an open-access publication distributed under the terms of the Creative Commons Attribution license.

Table 3. Likert Responses for Retrospective Pre- and Postworkshop Confidence in Identifying Microaggressions

Confidence in Identifying Microaggressions[a]	Preworkshop *n*	Postworkshop *n*
Very confident	7	24
Confident	41	49
Neutral	23	6
Unconfident	9	1
Total	80	80

[a]Participants rated their perceived confidence levels on a 5-point Likert scale (1 = *very unconfident*, 5 = *very confident*).

deidentified and anonymously submitted cases from our institution, emphasizes psychological safety, and has clear reproducibility.

Our simulation cases depict diverse roles for both aggressors and victims, including microaggressions from patients and families toward providers, providers toward patients, faculty toward trainees and staff, and peers toward each other. The simulation enables facilitators to role-play as both aggressors and victims, fostering cognitive empathy to promote compassionate and accountable communication. The simulation case list continues to evolve as more sessions are conducted and more cases are submitted. Unique to our curriculum, this has the potential to increase local relevance through the use of within-institution cases. As there is no defined case series, the limit of unique simulations is endless and stagnation is unlikely. The ability to submit cases for inclusion in future iterations of our curriculum may also serve to empower individuals who seek action and acknowledgment following their own experiences or observations.

We utilized palliative-care communication tools derived from simulation-based approaches to difficult conversations, allowing participants to pause for input or to rehearse their conversational approach prior to reengaging in the simulation.[25] Collaborative brainstorming and validation of the challenging nature of upstanding can then be applied to subsequent simulation conversations. This approach is integral to fostering a psychologically safe environment, specifically creating a low-stakes environment where participants can hear others' thoughts and validate challenges inherent to upstanding in real-world scenarios. Similar to previous studies,[10,12-14,18] participants in our curriculum had the ability to practice nuanced application of a general framework, rather than a prescriptive or algorithmic approach, which is more realistic and similar to challenges that arise in a clinical setting.

Psychological safety is essential for fostering a growth mindset and preventing retraumatization.[11] Throughout the workshops, we remained committed to fostering psychological safety and self-care, especially for those at risk of retraumatization due to the course content. However, the fact that 2% of participants denied feeling psychologically safe is a concern, and we cannot rule out the possibility that retraumatization may have occurred for the nearly 19% of respondents who did not answer the question about psychological safety. Of note, the survey question assessing psychological safety was written as an optional free-text response, to ensure that no participant felt forced to respond. We hoped that participants who took time to give optional free-text insight would help us improve the quality of the workshop, but it could have reduced the response rate. Among those who did not respond to the psychological safety question, they also omitted answers to other free-text questions. Of those respondents who left the psychological safety question blank, 13% ($n = 2$) said their comfort in speaking up after witnessing microaggressions did not change, and 87% ($n = 13$) said that their comfort increased.

Regardless of the reason for lack of participant response, we worry that at least some portion of the nonrespondents could have experienced harm. Another study, which used standardized patients, demonstrated a high degree of psychological safety (100%) in a smaller cohort;[10] this approach might be another way to minimize retraumatization for programs with adequate funding for these roles.

While it is difficult to delineate the most significant contributions to a psychologically safe environment, we believe that our emphasis on the universality of implicit bias, combined with the facilitators' willingness to demonstrate vulnerability by sharing personal shortcomings as upstanders, were key elements that enhanced psychological safety. Our experience was that almost all participants came to the session with the intention to learn and grow, but that harmful comments may still occur. When harms were perpetuated during the session, we modeled use of workshop tools in small-group settings without shaming. Whenever facilitators provided feedback to trainees about potential harms, facilitators shared their own experiences of unintentionally perpetuating harm and suggestions for alternative language. At the end of the session, we provided contact information for local mental health resources, and facilitators offered to privately debrief with any participant. We strongly recommend having at least one faculty mentor present for each workshop, given the vulnerable nature of the content.

This workshop increased participants' confidence in identifying microaggressions and their likelihood of speaking up when witnessing microaggression in clinical settings. Notably, no

Copyright © 2025 Hayward et al. This is an open-access publication distributed under the terms of the Creative Commons Attribution license.

participants reported decreased confidence or reduced likelihood of speaking up; the majority experienced improvement in both areas: 52% in identifying microaggressions, and 54% in confidence in speaking up. These findings suggest that this framework could be effectively used by other groups to achieve similar outcomes. Since we do not employ a standardized case series, repeating these workshops with the same participants should enhance their existing skills. One of the strengths of our framework is its ability to address microaggressions and facilitate difficult conversations specific to our institution. Thus, when implemented elsewhere, the framework can be adapted to address the unique issues relevant to other settings.

Through 4 years of project development, involving more than 700 participants, several key lessons have emerged. Delivering the content within a 60-minute session presented an ongoing challenge, with limited time available for practicing simulation cases and debriefing. In their feedback, participants indicated that if more time had been allocated, it would have made introverted participants feel more comfortable becoming engaged. Ideally, a 90–120-minute session would provide a more relaxed experience for both facilitators and participants. Nevertheless, we found that scheduling 60-minute workshops was more manageable and still valuable when longer sessions were not feasible. To accommodate time constraints, later iterations of our presentation focused less on didactic definitions and more on simulations and debriefing. An ideal facilitator-to-participant ratio is, in our experience, no more than 10–15 participants per facilitator. Large-group psychological safety and comfort sharing could be impacted by having groups larger than 20–25, but we have administered this workshop in larger groups of more than 100 participants, provided that small-group facilitator-to-participant ratios could be maintained. Participants appreciated the relevance of the simulation cases to their experiences.

This study has several limitations. First, while our survey was reviewed by experts in survey design at our institution, it was not validated or piloted. Second, this curriculum evaluation was conducted at a single, predominantly White institution in the Intermountain West, which may limit the applicability of our findings to other geographic regions or more diverse institutions. Third, we cannot clarify the reason for lack of response to our question about psychological safety for nearly 19% of participants. Additionally, we faced significant challenges in obtaining follow-up data, which significantly limits our ability to track behavior change over time. Further, self-assessments of confidence to upstand, rather than clinically observed behaviors, may not accurately reflect real-life responses to microaggressions. Finally, our facilitator pool consists of trainees and faculty who do not receive salary support, which may limit the sustainability and scalability of the program.

We continue to receive requests across our institution to present this workshop. This necessitates tailoring the simulation cases and content to specific areas of practice, and we have been unable to meet the demand with our current volunteer staffing model. Next steps for this project involve training additional facilitators, particularly beyond the physician population. With increased numbers of workshops, we will access a larger population, which will allow us to further evaluate the efficacy of our workshop. The ability to track behavior change longitudinally is another area of focus. Funding to encourage participants to complete follow-up surveys could improve participation. Finally, as highlighted in other studies, dedicated faculty support for diversity, equity, and inclusion efforts should be addressed across medical institutions.[35]

Implementing a case-based simulation workshop built on real clinical scenarios can be done in a psychologically safe environment that fosters upstanding. Participants who engage in the Speak Up! Simulation Workshop are better at identifying microaggressions and are more likely to intervene when witnessing such incidents in clinical settings. The curriculum is adaptable and repeatable, with deidentified simulation cases tailored to each institution, suggesting that our framework could be beneficial to other groups aiming to achieve similar outcomes.

Appendices

A. Speak Up! Simulation Workshop - Template.pptx

B. Facilitator Guide and Agenda.docx

C. Postworkshop Survey.docx

D. Participant Speak Up! Guide.docx

E. Deidentified Microaggression Case Bank.pptx

All appendices are peer reviewed as integral parts of the Original Publication.

Nathaniel E. Hayward, MD, MSc: Fellow, Department of Pediatrics, Pediatric Critical Care Program, Spencer Fox Eccles School of Medicine at the University of Utah; ORCID: https://orcid.org/0000-0001-9085-981X

Amanda J. McLearn-Montz, MD: Resident, Department of Pediatrics, Triple-Board Residency Program, Spencer Fox Eccles School of Medicine at the University of Utah

Copyright © 2025 Hayward et al. This is an open-access publication distributed under the terms of the Creative Commons Attribution license.

Katherine Amano, MD, MA: Resident, Department of Pediatrics, Triple-Board Residency Program, Spencer Fox Eccles School of Medicine at the University of Utah

Quang-Tuyen Nguyen, MD: Associate Professor, Department of Pediatrics, and Vice Chair for Health Equity, Respect, and Opportunity, Spencer Fox Eccles School of Medicine at the University of Utah

Rebecca Purtell, MD: Associate Professor, Department of Pediatrics, Division of Pediatric Hospital Medicine, Spencer Fox Eccles School of Medicine at the University of Utah

Katie Gradick, MD, MHS: Assistant Professor, Division of Palliative Care, Department of Pediatrics, Spencer Fox Eccles School of Medicine at the University of Utah

Reena P. Tam, MD: Associate Professor, Division of Pediatric Hospital Medicine, Department of Pediatrics, Spencer Fox Eccles School of Medicine at the University of Utah

Acknowledgments
The authors would like to acknowledge Gregory J. Stoddard, MBA, MPH, for guidance with the statistical analysis of the project data.

Disclosures
None to report.

Funding/Support
None to report.

Ethical Approval
The University of Utah Institution Institutional Review Board reviewed this project.

Disclaimer
The views and opinions expressed in the submitted article do not represent an official position of the University of Utah.

References

1. Greenwald AG, Banaji MR. Implicit social cognition: attitudes, self-esteem, and stereotypes. *Psychol Rev*. 1995;102(1):4-27. https://doi.org/10.1037/0033-295X.102.1.4
2. Pierce CM. Black psychiatry one year after Miami. *J Natl Med Assoc*. 1970;62(6):471-473.
3. Sue DW, Capodilupo CM, Torino GC, et al. Racial microaggressions in everyday life: Implications for clinical practice. *Am Psychol*. 2007;62(4):271-286. https://doi.org/10.1037/0003-066X.62.4.271
4. Bazargan M, Cobb S, Assari S. Discrimination and medical mistrust in a racially and ethnically diverse sample of California adults. *Ann Fam Med*. 2021;19(1):4-15. https://doi.org/10.1370/afm.2632
5. Lund S, D'Angelo JD, Jogerst K, Warner SG, Busch R, D'Angelo ALD. Revealing hidden experiences: gendered microaggressions and surgical faculty burnout. *Surgery*. 2022;172(3):885-889. https://doi.org/10.1016/j.surg.2022.04.032
6. Ackerman-Barger K, Goldin P, Jacobs NN, Johnson C, London M, Boatright D. Effect of microaggressions on medical residents' program satisfaction and depression. *J Best Pract Health Prof Divers*. 2022;15(1):1-14. https://www.jstor.org/stable/27211173?seq=1
7. Ahmad SR, Ahmad TR, Balasubramanian V, Facente S, Kin C, Girod S. Are you really the doctor? Physician experiences with gendered microaggressions from patients. *J Womens Health (Larchmt)*. 2022;31(4):521-532. https://doi.org/10.1089/jwh.2021.0169
8. Hu YY, Ellis RJ, Hewitt DB, et al. Discrimination, abuse, harassment, and burnout in surgical residency training. *N Engl J Med*. 2019;381(18):1741-1752. https://doi.org/10.1056/NEJMsa1903759
9. Grover A, Appelbaum N, Santen SA, Lee N, Hemphill RR, Goldberg S. Physician mistreatment in the clinical learning environment. *AM J Surg*. 2020;220(2):276-281. https://doi.org/10.1016/j.amjsurg.2019.11.038
10. Srinivasan S, Evaristo CE, Ali H, et al. Simulation training to interrupt microaggressions. *Acad Med*. 2024;99(12)(suppl): S68-S72. https://doi.org/10.1097/acm.0000000000005868
11. Grailey KE, Murray E, Reader T, Brett SJ. The presence and potential impact of psychological safety in the healthcare setting: an evidence synthesis. *BMC Health Serv Res*. 2021;21(1):773. https://doi.org/10.1186/s12913-021-06740-6
12. Torres MB, Salles A, Cochran A. Recognizing and reacting to microaggressions in medicine and surgery. *JAMA Surg*. 2019; 154(9):868-872. https://doi.org/10.1001/jamasurg.2019.1648
13. Fraley L, Sheffield V, Houchens N. Enhancing an experience-based patient-initiated mistreatment training. *Acad Med*. 2024;99(12)(suppl):S80-S86. https://doi.org/10.1097/acm.0000000000005870
14. Fernandez D, Gorgens S, McCann-Pineo M, Sperandeo M, Cassara M, Moadel T. Simulation vs RISE UP: A comparative study of approaches for teaching emergency medicine trainees how to manage microaggressions. *Acad Med*. 2024; 99(12)(suppl):S73-S79. https://doi.org/10.1097/acm.0000000000005869
15. Acholonu RG, Cook TE, Roswell RO, Greene RE. Interrupting microaggressions in health care settings: a guide for teaching medical students. *MedEdPORTAL*. 2020:16;10969. https://doi.org/10.15766/mep_2374-8265.10969
16. York M, Langford K, Davidson M, et al. Becoming active bystanders and advocates: teaching medical students to respond to bias in the clinical setting. *MedEdPORTAL*. 2021;17:11175. https://doi.org/10.15766/mep_2374-8265.11175
17. Walker VP, Hodges L, Perkins M, Sim M, Harris C. Taking the VITALS to interrupt microaggressions. *MedEdPORTAL*. 2022;18:11202. https://doi.org/10.15766/mep_2374-8265.11202

Copyright © 2025 Hayward et al. This is an open-access publication distributed under the terms of the Creative Commons Attribution license.

18. Sotto-Santiago S, Mac J, Duncan F, Smith J. "I didn't know what to say": responding to racism, discrimination, and microaggressions with the OWTFD approach. *MedEdPORTAL*. 2020;16:10971. https://doi.org/10.15766/mep_2374-8265.10971

19. Stephens KC, Redman T, Williams R, Bandstra B, Shah R. Considering culture and conflict: a novel approach to active bystander intervention. *MedEdPORTAL*. 2023;19:11338. https://doi.org/10.15766/mep_2374-8265.11338

20. Hock LE, Barlow PB, Scruggs BA, et al. Tools for responding to patient-initiated verbal sexual harassment: a workshop for trainees and faculty. *MedEdPORTAL*. 2021;17:11096. https://doi.org/10.15766/mep_2374-8265.11096

21. Pang J, Navejar N, Sánchez JP. Mistreatment in residency: intervening with the REWIND communication tool. *MedEdPORTAL*. 2022;18:11245. https://doi.org/10.15766/mep_2374-8265.11245

22. Calardo SJ, Kou M, Port C, et al. Realizing inclusion and systemic equity in medicine: upstanding in the medical workplace (RISE UP)—an antibias curriculum. *MedEdPORTAL*. 2022;18:11233. https://doi.org/10.15766/mep_2374-8265.11233

23. Famouri ML, Hernandez S, Omlor RL, et al. Active bystander training: using standardized patient methodology to teach residents to navigate microaggressions in patient encounters. *MedEdPORTAL*. 2023;19:11298. https://doi.org/10.15766/mep_2374-8265.11298

24. Hill Weller L, Tang J, Chen R, Boscardin C, Ehie O. Tools for addressing microaggressions: an interactive workshop for perioperative trainees. *MedEdPORTAL*. 2023;19:11360. https://doi.org/10.15766/mep_2374-8265.11360

25. Wittkower D, Bryan J, Asghar-Ali A. Policies, recommendations and training to respond to patient microaggressions and hate speech aimed at healthcare professionals: a systematic review. *Eur Psychiatr*. 2021;64(suppl 1):S384-S384. https://doi.org/10.1192/j.eurpsy.2021.1029

26. Yeheskel A, Rawal S. Exploring the 'patient experience' of individuals with limited English proficiency: a scoping review. *J Immigrant Minority Health*. 2019;21(4):853-878. https://doi.org/10.1007/s10903-018-0816-4

27. Flores G. Families facing language barriers in healthcare: when will policy catch up with the demographics and evidence? *J Pediatr*. 2014;164(6):1261-1264. https://doi.org/10.1016/j.jpeds.2014.02.033

28. Oluo I. *So You Want to Talk about Race*. Seal Press; 2018.

29. Markin A, Cabrera-Fernandez DF, Bajoka RM, et al. Impact of a simulation-based communication workshop on resident preparedness for end-of-life communication in the intensive care unit. *Crit Care Res Pract*. 2015;2015:534879. https://doi.org/10.1155/2015/534879

30. Davitadze M, Ooi E, Ng CY, et al. SIMBA: using Kolb's learning theory in simulation-based learning to improve participants' confidence. *BMC Med Educ*. 2022;22(1):116. https://doi.org/10.1186/s12909-022-03176-2

31. Kolb DA. *Experiential Learning: Experience as the Source of Learning and Development*. Prentice Hall; 1984.

32. Johnston S, Coyer FM, Nash R. Kirkpatrick's evaluation of simulation and debriefing in health care education: a systematic review. *J Nurs Educ*. 2018;57(7):393-398. https://doi.org/10.3928/01484834-20180618-03

33. Artino AR Jr, La Rochelle JS, Dezee KJ, Gehlbach H. Developing questionnaires for educational research: AMEE Guide No. 87. *Med Teach*. 2014;36(6):463-474. https://doi.org/10.3109/0142159X.2014.889814

34. Bowker AH. A Test for symmetry in contingency tables. *J Am Stat Assoc*. 1948;43(244):572-574. https://doi.org/10.1080/01621459.1948.10483284

35. Rubenstein J, Rahiem S, Nelapati SS, Arevalo Soriano T, Tatem A. Discrimination 911: a novel response framework to teach bystanders to become upstanders when facing microaggressions. *Acad Med*. 2023;98(7):800-804. https://doi.org/10.1097/ACM.0000000000005171

Received: January 3, 2025
Accepted: July 17, 2025
Published: August 29, 2025

Copyright © 2025 Hayward et al. This is an open-access publication distributed under the terms of the Creative Commons Attribution license.
